# Repetition Suppression for Mirror Images of Objects and Not Braille Letters in the Ventral Visual Stream of Congenitally Blind Individuals

**DOI:** 10.1523/ENEURO.0002-25.2025

**Published:** 2026-01-09

**Authors:** Maksymilian Korczyk, Katarzyna Rączy, Marcin Szwed

**Affiliations:** ^1^Department of Psychology, Jagiellonian University, Kraków 30-060, Poland; ^2^Institute of Psychology, University of Hamburg, Hamburg 20146, Germany

**Keywords:** Braille, breaking mirror invariance, congenitally blind individuals, mirror invariance, reading, shape recognition

## Abstract

Mirror invariance is the cognitive tendency to perceive mirror-image objects as identical. Mirrored letters, however, are distinct orthographic units and must be identified as different despite having the same shape. Consistent with this phenomenon, a small, localized region in the ventral visual stream, the Visual Word Form Area (VWFA), exhibits repetition suppression to both identical and mirror pairs of objects but only to identical, not mirror, pairs of letters ( Pegado et al., 2011), a phenomenon named mirror invariance “breaking”. The ability of congenitally blind individuals to “break” mirror invariance for pairs of mirrored Braille letters has been demonstrated behaviorally ( de Heering et al., 2018, Korczyk et al., 2024). However, its neural underpinnings have not yet been investigated. Here, in an fMRI repetition suppression paradigm, congenitally blind individuals (8 males and 10 females) recognized pairs of everyday objects and Braille letters in identical (“p” and “p”), mirror (“p” and “q”), and different (“p” and “z”) orientations. We found repetition suppression for identical and mirror pairs of everyday objects in the parietal and ventral-lateral occipital cortex, indicating that mirror-invariant object recognition engages the ventral visual stream in tactile modality as well. However, repetition suppression for identical but not mirrored pairs of Braille letters was found not in the VWFA, but in broad areas of the left parietal cortex and the lateral occipital cortex. These results suggest that reading-related orthographic processes in blind individuals depend on different neural computations than those of the sighted.

## Significance Statement

Mirror invariance is a perceptual bias to recognize mirrored objects as identical. Letters constitute a unique category of objects: for example, “b” and “d” share identical shape yet must be identified as distinct entities to enable efficient reading. Here, we investigated the neural underpinnings of tactile mirror invariance in congenitally blind individuals and whether it was affected by tactile reading acquisition. We found that the parietal, occipital, and ventral visual regions were engaged in mirror-invariant tactile object recognition, indicating that this perceptual bias extends beyond the visual modality. Moreover, we found that unlike in the sighted, it was the parietal and lateral occipital cortex that showed neural signatures of breaking mirror invariance for Braille letters in congenitally blind individuals, demonstrating, that following congenital visual deprivation, neural computations can be repurposed to meet novel task requirements.

## Introduction

Mirror invariance is an automatic predisposition of the visual system to recognize objects reflected on both vertical and horizontal axes as identical (e.g.,Corballis and Beale, 1976; Tarr and Pinker, 1989). The neural underpinnings of this phenomenon were demonstrated in both macaque monkeys (Rollenhagen and Olson, 2000) and humans (Dehaene et al., 2010; Pegado et al., 2011) using fMRI repetition suppression studies. Repetition suppression—known as well as an fMRI priming effect—refers to a decrease in neural response to a target stimulus (e.g., the word “SHEEP” or a picture of a sheep) when it is preceded by an identical (that is, sharing the same identity) stimulus (referred to as a prime) relative to when it is preceded by an unrelated and/or different stimulus. In the context of object recognition, mirror invariance is reflected in repetition suppression of the neural response to a target object in regions of the ventral visual stream when it is preceded by its identical or mirrored image, even though the retinal projections for this object and its mirror reflection differ significantly (Dehaene et al., 2010; Dilks et al., 2011; Pegado et al., 2011). However, mirror invariance becomes a challenge when children begin to read, as mirrored letters of many scripts, such as the Latin “b” and “d,” have to be discriminated as different (Cornell, 1985; Ahr et al., 2016; Fischer and Koch, 2016) to enable reading. Once mirror invariance for letters is “broken,” that is, mirrored letters such as “b” and “d” are recognized as distinct orthographic units despite having identical shapes, the Visual Word Form Area (VWFA)—a region crucial for reading (Dehaene and Cohen, 2011; Zhan et al., 2023)—shows repetition suppression for mirrored pairs of objects but not for pairs of mirrored letters (Dehaene et al., 2010; Pegado et al., 2011).

Importantly, the Braille alphabet also includes mirror-letter pairs and in fact, contains more of them than the Latin alphabet. As a consequence, similar to their sighted peers, blind children struggle with mirror letters when they first start learning to read in Braille (Millar, 2004). In line with this, De Heering and colleagues (2018) demonstrated that congenitally blind individuals “break” mirror invariance for Braille letters too, similarly to sighted readers: judging the shape of two mirrored Braille letters as having the same shape is more demanding than doing so for objects. In another behavioral study (Korczyk et al., 2024), we replicated these findings by demonstrating high expertise of blind individuals with Braille letters, particularly in recognizing different orientations of mirror letters. However, we did not find significant difficulties when participants judged the shape of two left–right oriented Braille letters as having the same shape. We speculated that blind Braille readers might exhibit greater selective attention, allowing them to filter out task-irrelevant information more effectively (Korczyk et al., 2024). Nevertheless, both studies highlighted the substantial perceptual expertise of blind individuals in processing Braille letters and thus suggested necessary neural adaptations to Braille reading. However, whether these adaptations mirrored the neural network organization of sighted readers or alternatively engaged different cortical regions has not yet been investigated. While tactile Braille reading has been shown to activate the ventral occipital temporal cortex (vOTC) in a task-specific manner (Reich et al., 2011; Amedi et al., 2017, Rączy et al., 2019), it was shown to recruit additional cortical areas as well, including primary visual areas and parietal cortices (presumably due to tactile nature of Braille; Saccone et al., 2024)—suggesting a more distributed and potentially modality-specific neural architecture whose functional contributions to Braille reading remain incompletely understood.

**Figure 1.**
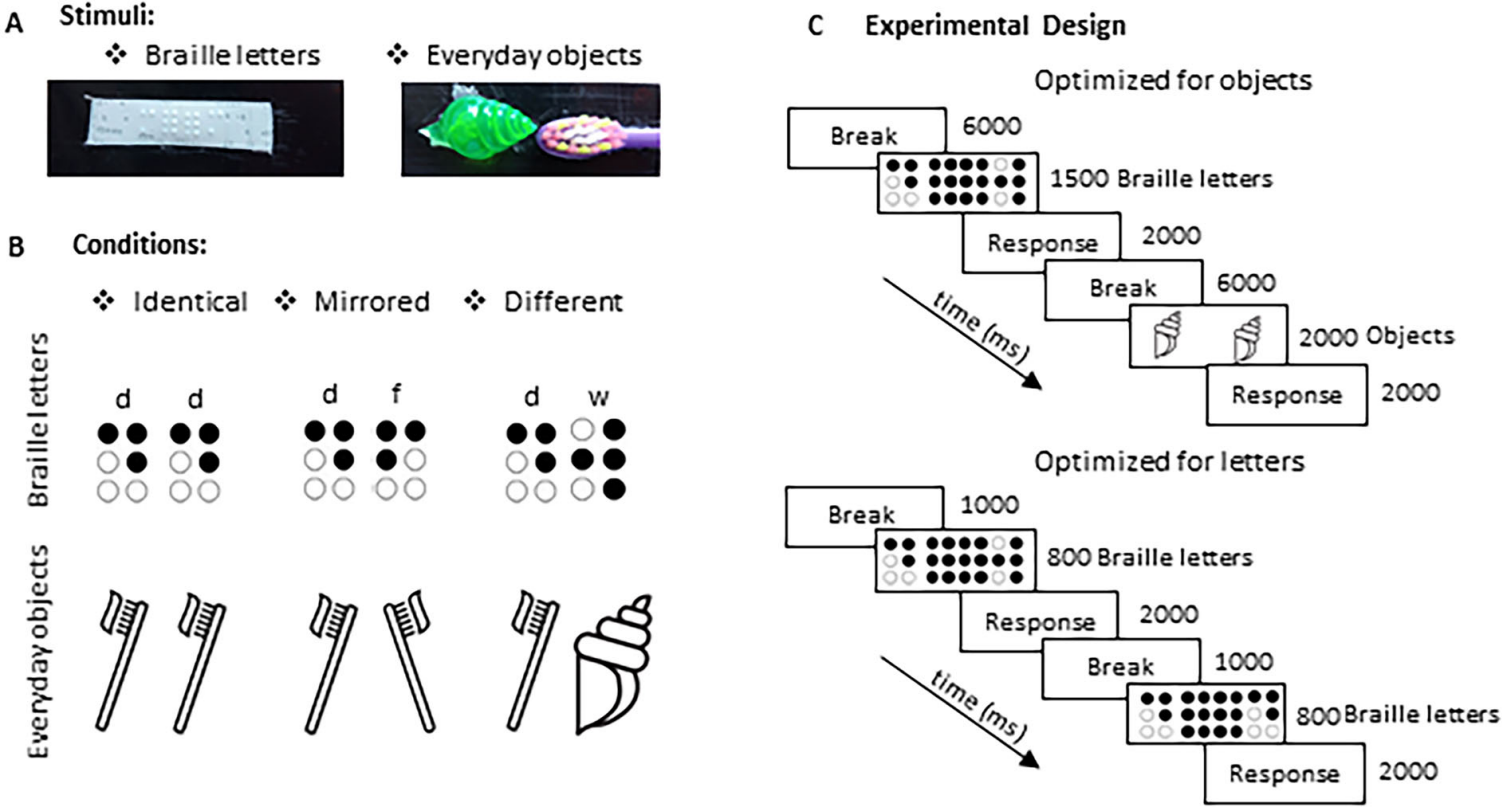
***A***, Tactile stimuli used in the first fMRI experiment: Braille letters and everyday objects. Both Braille letters and everyday objects were presented on a conveyor belt. ***B***, In both fMRI experiments, all stimuli were presented under identical, mirrored, and different conditions. ***C***, Experimental design. An fMRI priming paradigm was implemented. In the first fMRI experiment, Braille letters were presented for 1,500 ms and objects for 2,000 ms. This presentation timing was optimal for objects but not for Braille letters. In the second fMRI experiment, the focus was on Braille letters, which were presented for 800 ms, optimal presentation time for Braille letters. For additional information, see Extended Data Figure 1-1.

Here, we investigated the neural underpinnings of mirror-letter discrimination in Braille reading with an fMRI adaptation paradigm. This approach allowed us to examine the neural loci of “breaking” of mirror invariance for Braille letters and, in turn, to investigate the neural mechanisms supporting tactile reading in the absence of vision. Participants were presented with two categories of haptic stimuli: everyday objects and Braille letters (Fig. 1*A*). All stimulus categories were presented sequentially in three different prime–target pairs: identical (“b” and “b”), mirrored (“b” and “d”), or different (“b” and “g”; Fig. 1*B*). We reasoned that if we found repetition suppression for identical and mirrored pairs of objects and identical but not mirrored pairs of Braille letters in a given brain region, it would provide evidence that this brain region is the locus of letter-recognition in blind individuals. If that brain region is found in the location of the sighted VWFA, it would support the VWFA being a task-specific reading region of the brain (Amedi et al., 2017), independent of the input modality, and not a general language area (Bedny, 2017). However, if we found this pattern of results beyond the VWFA in, e.g., the lateral occipital cortex (LOC), which has been suggested to process shape information independent of the input modality (Hannagan et al., 2015), or parietal cortex—suggested to be engaged in letter-identity processing in Braille reading (Tian et al., 2023, Liu et al., 2023), it would suggest that blind individuals’ reading system notably differs from the one of sighted individuals.

10.1523/ENEURO.0002-25.2025.f1-1 Figure 1-1 (A) The conveyor belt was specially designed for this study to be fMRI-compatible. (B) A picture of the belt with plastic plates containing pairs of stimuli and spacers – gaps between each stimuli plate intended to avoid any possible mistakes. (C) The device was placed above the participants’ thighs on their reading side. The researcher moved the chain and remained at the end of the device. (D)The participants’ reading hand was located in a special aperture, allowing them to touch only one pair of stimuli at a time. Download Figure 1-1, TIF file.

## Materials and Methods

In the current study, we conducted two fMRI experiments. Experiment 1 was optimized for the perception of 3D everyday objects, featuring longer stimulus presentation times. The total duration of this experiment was ∼1.5 h. Experiment 2 was optimized for the perception of Braille letters, using shorter stimulus presentation times (Fig. 1*C*). The total duration of this experiment was ∼1 h.

### Participants

Eighteen congenitally blind native Polish speakers participated in the first fMRI experiment (7 females, mean age: 31.9 years, SD = 5.3). Another group of 18 congenitally blind Polish speakers took part in the second fMRI experiment (10 females, mean age: 33.2 years, SD = 6.14). Fourteen of the 18 participants completed both fMRI experiments. Congenitally blind individuals represent a rare and hard-to-find clinical population. This sample size is comparable with—or larger than—recent fMRI studies with blind individuals, such as Kanjlia, Feigenson, and Bedny (2021) (17 participants), Vetter et al. (2021) (8 participants), and Bola et al. (2023) (9 participants).

In the first fMRI study, the main causes of blindness among participants were retinopathy of prematurity (*n* = 12), optic nerve atrophy (*n* = 4), and other causes (*n* = 2; for details, see Table 1). Eleven of the 18 participants were completely blind, while the remaining had primitive light sensitivity. In the second fMRI study, the main causes of blindness were retinopathy of prematurity (*n* = 13), optic nerve atrophy (*n* = 3), and other causes (*n* = 2; see Table 2 for details). Thirteen participants were completely blind, while the rest had primitive light sensitivity.

**Table 1.** Demographic information for congenitally blind individuals (Experiment 1)

Participant	Age (years)	Gender	Cause of blindness	Reading hand
1	45	Female	Retinopathy of prematurity	Right (index finger)
2	32	Female	Retinopathy of prematurity	Right (index finger)
3	32	Female	Retinopathy of prematurity	Left
4	31	Male	Retinopathy of prematurity	Right
5	35	Male	Glaucoma	Right
6	24	Male	Atrophy of the optic nerve	Left (index finger)
7	30	Female	Atrophy of the optic nerve	Right
8	34	Male	Genetic	Left
9	30	Male	Atrophy of the optic nerve	Right
10	28	Female	Retinopathy of prematurity	Right
11	27	Male	Retinopathy of prematurity	Right
12	31	Female	Atrophy of the optic nerve	Right (index finger)
13	25	Male	Retinopathy of prematurity	Left
14	26	Male	Retinopathy of prematurity	Left
15	36	Male	Retinopathy of prematurity	Left
16	39	Male	Retinopathy of prematurity	Left
17	39	Female	Retinopathy of prematurity	Left (index finger)
18	31	Male	Retinopathy of prematurity	Left

**Table 2.** Demographic information for congenitally blind individuals (Experiment 2)

Participant	Age (Years)	Gender	Cause of Blindness	Reading Hand
1	45	Female	Retinopathy of prematurity	Right (index finger)
2	32	Female	Retinopathy of prematurity	Right (index finger)
3	32	Female	Retinopathy of prematurity	Left
4	40	Female	Retinopathy of prematurity	Left
5	35	Male	Glaucoma	Right
6	24	Male	Atrophy of the optic nerve	Left (index finger)
7	30	Male	Atrophy of the optic nerve	Right
8	35	Female	Genetic	Right (index finger)
9	37	Female	Retinopathy of prematurity	Right
10	28	Female	Retinopathy of prematurity	Right
11	27	Male	Retinopathy of prematurity	Right
12	31	Female	Atrophy of the optic nerve	Right (index finger)
13	25	Male	Retinopathy of prematurity	Left
14	26	Male	Retinopathy of prematurity	Left
15	36	Male	Retinopathy of prematurity	Left
16	39	Female	Retinopathy of prematurity	Left (index finger)
17	45	Female	Retinopathy of prematurity	Left
18	31	Male	Retinopathy of prematurity	Left

All participants were blind from birth and had never experienced patterned vision. None reported any additional sensory or motor disabilities, neurological conditions, or psychiatric disorders.

All fMRI procedures were conducted in accordance with the Jagiellonian University Ethics Committee's regulations. Participants were reimbursed for participating in the study.

### Stimuli

In the first fMRI experiment, two categories of stimuli were used: everyday objects [14: the lid of a tic-tac mint box, ½ adhesive tape, measuring spoon (7.5 mm), toothbrush, pen lid, bag clips, a measuring spoon (5 mm), three types of acrylic plastic seashells for aquariums, four pills in a blister pack, ear stick, comb, and straw] and Braille letters (8: d, e, h, m, p, r, y, and z; Fig. 1*A*). All stimuli were presented in pairs under three conditions: identical (identical pairs of stimuli presented in the same orientation), mirrored (identical pairs of stimuli presented in mirror symmetry), and different (different pairs of stimuli presented in different orientations; Fig. 1*B*). All stimuli were glued to a plastic plate (8 × 3 cm). A 1 cm distance between pairs of everyday objects was maintained. All everyday objects were purchased from common supermarkets, drugstores, or stationery shops. Before the fMRI experiment, we conducted pilot and behavioral experiments (Korczyk et al., 2024) to verify whether the participants were able to accurately and quickly recognize the everyday objects, later used in both behavioral and fMRI experiments. This allowed us to select 14 stimuli that all participants correctly recognized in <2 s.

Braille letter stimuli were printed on an RL-350 Braille labeler on Reizen vinyl label tape. Between pairs of Braille letters, we added two six-dot characters, and these stimulus prints were then glued in the center of the 8 × 3 cm plastic plate.

In the second fMRI experiment, the primary focus was on Braille letters, and therefore, we presented the participants with all 32 letters of the Polish alphabet displayed in pairs in three conditions: identical, mirrored, and different (see above). Of these, nine letters have mirror equivalents, which result in 18 letters in total with mirror counterparts, a consequence of the limited number of dot combinations available on the six-dot Braille matrix. The mirror reflections of the remaining letters typically correspond to other meaningful signs, such as those used in musical or chemical notation, or punctuation. As a result, in the following experiment, only pairs of reversible letters were used in the mirror condition, while in the different condition, the two letters in each pair differed both in shape and identity.

In the letter condition of both Experiments, we used two six-dot Braille characters as a separator for two reasons: First, the six-dot character is the tactile equivalent of a “#” sign in the visual alphabet and carries no orthographic meaning. It has been widely used as a tactile control in previous studies (Rączy et al., 2019; Korczyk et al., 2024), as it stimulates the same spatial area with a consistent dot pattern, without conveying linguistic information. This approach ensures that any tactile input between letters remains constant across trials, helping to control for potential confounds related to differences in sensory stimulation, while preserving the sequential exploration typical of Braille reading. Second, using a consistent tactile marker helped participants reliably perceive the beginning and end of each letter sequence, minimizing ambiguity that could arise from leaving empty spaces, which are harder to detect in tactile exploration. Notably, as shown by our behavioral results and prior studies (de Heering et al., 2018; Korczyk et al., 2024), the six-dot separator does not significantly interfere with letter processing or the neural mechanisms underlying mirror invariance breaking.

### Procedure

In the first fMRI experiment, everyday objects and Braille letters were presented on a conveyor belt designed for tactile stimuli. We used Presentation software (Neurobehavioral Systems; https://www.neurobs.com/) to deliver auditory cues that controlled the presentation of tactile stimuli. Each cue instructed the experimenter to move the conveyor belt to the next set of stimuli. The conveyor belt was custom-designed to be fMRI-compatible (Neuro Device) for this study (Extended Data Fig. 1-1*A–D*). It featured 80 slots for attaching plastic plates with stimuli. A total of 39 plastic plates, used across all runs, were attached to every second slot. To minimize potential errors, spacers (gaps) were introduced between each stimulus plate (Extended Data Fig. 1-1*B*).

The device was positioned above the participants’ thighs (Extended Data Fig. 1-1*C*). Their reading hand was placed in a designated aperture, allowing them to touch only one pair of stimuli at a time (Extended Data Fig. 1-1*D*). Participants read Braille and recognized everyday objects using their preferred Braille reading hand. Throughout all runs, the same researcher controlled the movement of the conveyor belt. The precise timing for when the conveyor belt was to be moved was delivered to the experimenter via auditory cues through headphones. This procedure allowed us to maintain strict control over stimulus timing despite the manual delivery mechanism.

The first fMRI experiment consisted of seven runs, with participants spending 90 min inside the scanner to complete all tasks. Across all runs, we presented 138 pairs of everyday objects and 135 pairs of Braille letters. In odd-numbered runs (1, 3, 5, and 7), participants were presented with 18 pairs of Braille letter stimuli (6 pairs per condition) and 21 pairs of everyday objects (7 pairs per condition). In even-numbered runs (2, 4, and 6), they were presented with 21 pairs of Braille letter stimuli (7 pairs per condition) and 18 pairs of everyday objects (6 pairs per condition). Each trial began with an auditory cue indicating the type of stimuli to be presented, followed by a 200 ms beep at a frequency of 4,000 Hz. This beep signaled the start of either a Braille letter trial (presented for 1,500 ms) or an everyday object trial (presented for 2,000 ms). A second auditory cue, a 200 ms beep at a frequency of 44,100 Hz, marked the end of the trial. To minimize top-down processing, we employed a catch-trial paradigm in which participants focused on recognizing either a seashell or the letter “M”. In Polish, the word for seashell (muszelka) starts with the letter “M”, making the task more intuitive for participants. Participants had 2,000 ms to indicate (by pressing a corresponding button) whether they recognized one seashell or one letter “M”, or two seashells or two letters “M”, respectively (Fig. 1*C*). Following this, there was a 6,000 ms break, allowing the researcher to advance the conveyor belt.

In the second fMRI experiment, stimuli were presented using Presentation software (Neurobehavioral Systems; https://www.neurobs.com/). Braille letters were displayed on an fMRI-compatible Braille display (Neuro Device; Debowska et al., 2013), similar to commercial Braille devices, featuring pneumatically driven Braille pins. This device can simultaneously present five Braille letters, allowing participants to read them as they would in regular Braille text. The Braille display was positioned on the participants’ thighs, on the side corresponding to their reading hand.

The second fMRI experiment consisted of five functional runs. In each run, we presented 108 pairs of Braille letters (36 pairs per condition), along with 16 “blank trials,” during which no auditory cue or tactile stimulus was presented on the Braille display for 2,800 ms (the same duration as the trial). Each trial began with an auditory cue (a 200 ms beep at a frequency of 1,500 Hz), signaling the presentation of Braille letters, which were displayed for 800 ms. Each pair of Braille letters was separated by a double six-dot character (which has no meaning in the Polish Braille alphabet). A second auditory cue (another 200 ms beep at 1,500 Hz) signaled the end of the trial. To minimize top-down effects, we followed previous studies in the visual and tactile domains (Binder et al., 2006; Vinckier et al., 2007; Raczy et al., 2019) and required the participants to focus more on perceptual aspects of Braille characters. Specifically, the participants were instructed to press one button if they recognized a pair of three-dot Braille letters, to press another button if they perceived only one three-dot Braille letter in a pair, and to refrain from pressing any button if no three-dot Braille letters were presented. They had 2,000 ms to respond (Fig. 1*C*), followed by a 1,000 ms break.

Additionally, there was a localizer scan in which we presented the participants with 96 Braille-character stimuli (3 Braille letters separated by a six-dot character), along with 11 “blank trials,” during which no auditory cue or tactile stimulus was presented on the Braille display for 2,800 ms (the same duration as the trial), and 28 trials where we presented 5 six-dot characters as a tactile control. Each trial began with an auditory cue (a 200 ms beep at a frequency of 1,500 Hz), signaling the presentation of Braille letters, which were displayed for 800 ms.

### fMRI data acquisition

All fMRI data were acquired at the Małopolskie Centrum Biotechnologii in Kraków. Both fMRI experiments followed the same settings for functional and anatomical scans. Functional MRI scans were collected using an EPI sequence on a 3 T Siemens Skyra scanner equipped with a 64-channel head coil (flip angle, 70°; TR, 1,200 ms; TE, 27 ms; FOV, 240 mm; matrix size, 80 × 80). Forty-two contiguous axial slices were acquired, each with a thickness of 3.0 mm and an in-plane resolution of 3.0 × 3.0 mm^2^. We employed multiband imaging (MB factor = 2) to enhance the temporal resolution of fMRI data acquisition. For anatomical reference and spatial normalization, T1-weighted images were acquired using an MPRAGE sequence (208 slices; FOV, 250 mm; TR, 1,800 ms; TE, 2.37 ms; voxel size, 0.9 × 0.9 × 0.9 mm^3^).

### Behavioral data analysis

In both experiments, we implemented a catch-trial paradigm, allowing for accuracy measurement. Reaction times were not assessed, as neither the priming effect nor the effect of stimulus orientation would be observable in any of the tasks. Participants' ability to accurately recognize stimuli and their orientation was evaluated in a separate behavioral study (Korczyk et al., 2024) conducted with the largely overlapping participants.

### fMRI data analysis

All fMRI data were analyzed using the SPM12 software package (https://www.fil.ion.ucl.ac.uk/spm/software/spm12/). Data preprocessing steps included the following: (1) slice timing correction; (2) realignment of all EPI images to the first image; (3) coregistration of the anatomical image to the mean EPI image; (4) normalization of all images to MNI space; and (5) spatial smoothing with a 6 mm FWHM kernel. The hemodynamic response function (HRF) for main predictors (identical, mirror, and different pairs) and six estimated movement parameters as confound predictors were first modeled within a general linear model (GLM, Friston et al., 1997) for each participant. For the localizer scan, the HRF for main predictors (Braille letters and tactile control) and six estimated movement parameters as confound predictors were modeled within a GLM for each participant.

#### Whole-brain analysis

Next, for both everyday objects and Braille letters, we conducted the following comparisons: (1) mirror versus identical condition, (2) different versus identical condition, and (3) different versus mirror condition. Subsequently, we overlapped brain activation induced by two different comparisons: (1) different versus identical and different versus mirror conditions for everyday objects; (2) different versus identical and mirror versus identical conditions for Braille letters; and (3) different versus identical conditions in both everyday objects and Braille letters. We reasoned that if we found repetition suppression for identical and mirrored pairs of objects and identical but not mirrored pairs of Braille letters, as compared with different pairs, in a given brain region, it would provide evidence that this brain region processes objects in a mirror-invariant way and that this perceptual bias was “broken” for letters as a result of Braille alphabet acquisition.

Finally, we conducted a random-effects ANOVA analysis for the group. In the first experiment, we applied a voxel-wise threshold of *p* < 0.05 FWE and a cluster extent threshold of *p* < 0.05 FWE. In the second experiment, we used a voxel-wise threshold of *p* < 0.001 (uncorrected) and a cluster extent threshold of *p* < 0.05 FWE. To support the localization of the observed effects, we used a probabilistic atlas of the human brain implemented in the SPM Anatomy Toolbox 2.2b (Eickhoff et al., 2005).

#### ROI analysis and functional localizer

To identify the neural network engaged during tactile Braille reading and to ensure that we were examining regions reliably activated by this task, we included a localizer run in Experiment 2. In this localizer, we contrasted the activation induced by sequences of three Braille letters (each separated by a six-dot character) with sequences of five six-dot characters, which served as the basic tactile control stimulus. This contrast revealed activation across the parietal-dorsal-ventral stream (Extended Data Fig. 4-8).

A number of regions were a priori defined as areas of particular interest. These included the VWFA, due to its crucial role in orthographic processing in sighted individuals and a loci of breaking mirror invariance for letters, the parietal cortex—particularly the posterior parietal cortex (PPC)—which has been proposed to support letter-identity processing in Braille reading (Liu et al., 2023); primary visual cortex (V1), as it has been suggested to repurpose for higher-level semantic functions following blindness (Bedny, 2017); and the lateral occipital cortex (LOC), which has been argued to process geometry independently of sensory modality (Hannagan et al., 2015) and to encode both perceptual and sensory aspects of Braille characters in blind individuals (Haupt et al., 2024).

Additional to the latter ROIs (regions of interest), we created anatomical masks within the activated reading network in the localizer scan (see Extended Data Figs. 4-1, 4-2–4-7 for the statistical map in the corresponding condition) which overlapped with the regions reported for visual mirror invariance and tactile symmetry in sighted individuals (Aspell et al., 2010; Dehaene et al., 2010; Pegado et al., 2011; Kitada et al., 2014; Fujimoto et al., 2017) to enable more fine-grained investigation of their respective computational roles in tactile reading. This allowed us to determine which of these areas were specifically involved in the “breaking” of mirror invariance for Braille letters.

The MarsBaR 0.44 Toolbox (Brett et al., 2002; http://marsbar.sourceforge.net/) was used to conduct functionally guided ROI analyses. We examined signal changes induced by three different conditions (identical, mirror, and different) for everyday objects and Braille letters in both fMRI experiments. Anatomical masks for the left occipital-temporal and bilateral parietal areas were created using the SPM Anatomy Toolbox 2.2b (Eickhoff et al., 2005). The left occipital-temporal regions encompassed areas such as the following: (1) the primary visual cortex (V1 and V2, i.e., BA 17 and BA 18), (2) middle-temporal cortex [hOC5 (V5/MT+)], (3) ventral (V3v/V4) and (4) dorsal extrastriate cortex (hOC3d/hOC4d), (5) fusiform gyrus (Areas FG1, FG2, FG3, and FG4), and (6) lateral occipital cortex (extrastriate areas hOc4la and hOc4lp; Extended Data Fig. 4-1*A*). For the bilateral parietal cortex, we created masks for (1) the intraparietal sulcus (areas hIP1, hIP2, hIP3), (2) motor cortex (areas 4a and 4p), and (3) primary somatosensory cortex (areas 1, 2, 3a, 3b; Extended Data Fig. 4-1*B*).

Next, we extracted beta estimates from each voxel within the anatomical masks and averaged them across voxels for each subject and for each condition. The mean beta estimates were then entered into a repeated-measures ANOVA to assess the effect of orientation on regional activation. All statistical tests were corrected for multiple comparisons using the Bonferroni’s correction.

#### VWFA ROI

To probe for the repetition suppression effect within the location of the sighted VWFA—characteristic of mirror invariance breaking (i.e., repetition suppression to identical and mirror pairs of objects and only to identical but not mirror pairs of letters relative to totally different pairs), we employed two ROI definitions. First, following Rączy et al. (2019), we functionally defined the VWFA ROI in each participant as 30 (not necessarily contiguous) voxels with the highest *t* value in the Braille letters versus tactile control contrast (localizer of Experiment 2). Since in six participants <30 voxels were identified, in addition we decided to restrict the ROI to 10 most active voxels to ensure that no significant activation was omitted. As a result, 10 noncontiguous voxels with the highest *t* value in the Braille letters versus rest contrast were selected. Two participants had to be nevertheless excluded, as <10 voxels were identified. In both cases, the selection was done within the anatomically specified boundaries of the given region (Zmax = −10, Zmin = −25, Ymax = −70, Ymin = −40, Xmax = −60, Xmin = −30). Subsequently, for both, 30 voxels and 10 voxels, we extracted beta values from these voxels across the identical, mirror, and different conditions for both everyday objects (Experiment 1) and Braille letters (Experiment 2). These beta values were then averaged across the selected voxels, and repeated-measures ANOVAs were conducted on beta values for the three conditions: identical, mirror, and different. Separate analyses were conducted for everyday objects and Braille letters, accounting for the number of voxels included in the ROI definition (Fig. 5). Second, VWFA was defined as an 8 mm radius sphere with the canonical coordinates reported by Cohen et al. (2004; MNI: *x* = −41, *y* = 57, *z* = −16) and the analysis was done by the MarsBaR toolbox. Beta values were extracted from all voxels within this ROI for each participant, averaged across all voxels and then submitted to a repeated-measures ANOVA with three within-subject conditions: identical, mirror, and different (Fig. 5).

## Results

In the first fMRI study, participants achieved an accuracy of 73.5% (SD = 13.7%), while in the second study, their accuracy was 76.6% (SD = 15.0%). A paired *t* test revealed no significant difference in performance between the two experiments, *t*_(17)_ = −0.617, *p* = 0.545.

### Experiment 1—everyday objects and Braille letters

#### Whole-brain analysis

**Figure 2.**
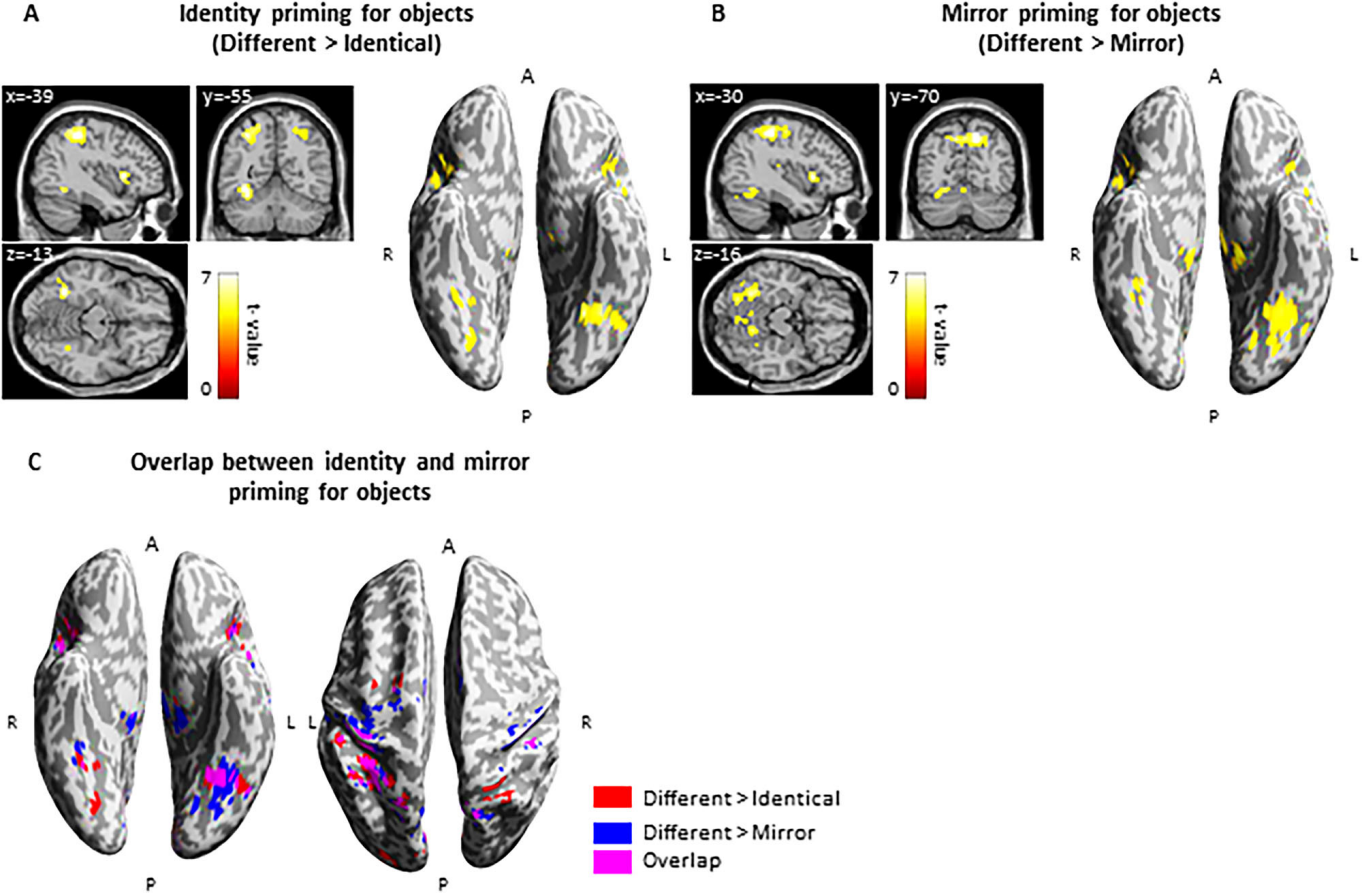
FMRI priming effects for tactile objects. ***A***, Identity priming: Objects induced bilateral effects in the dorsal parietal, posterior-medial frontal, and occipito-temporal regions. ***B***, Mirror priming: Objects evoked mirror priming in a wide area of the brain bilaterally, including the visual cortex, dorsal parietal, and posterior-medial frontal regions. ***C***, Overlap between identity and mirror priming for objects: We observed overlap in the left fusiform cortex (−33, −55, −19) and parietal cortex, including the intraparietal sulcus, motor cortex, and primary somatosensory cortex. However, mirror priming induced broader activation in this region compared with identity priming. Thresholds: ***A–C***, *p* < 0.05 FWE voxel-wise, *p* < 0.05 FWE cluster-wise. R, right; L, left; A, anterior; P, posterior. For additional information, see Extended Data Figures 2-1 and 2-2.

To probe for repetition suppression, we first contrasted everyday objects presented in different versus identical conditions (identity priming). We observed a repetition suppression effect bilaterally in a fronto-parieto-temporo-occipital network (Fig. 2*A*). Specifically, the activation difference was observed bilaterally in the dorsal parietal regions, including the left inferior parietal lobule gyrus (−39, −40, 50; *t* = 6.98, 367 voxels) and the right precuneus (45, 12, −70; *t* = 6.26, 45 voxels), as well as the superior parietal lobule (24, −52, 56; *t* = 5.85, 60 voxels), in the frontal regions, encompassing the left superior frontal gyrus (−24, −7, 53; *t* = 5.64, 31 voxels) and the right posterior-medial frontal gyrus (6, 11, 53; *t* = 5.79, 135 voxels), as well as in the occipitotemporal cortex, including the ventral visual stream with activations in the left fusiform gyrus (−33, −55, −10; *t* = 7.34, 75 voxels) and the right fusiform gyrus (27, −70, −7; *t* = 5.44, 14 voxels; Fig. 2*A*, Extended Data Fig. 2-1).

10.1523/ENEURO.0002-25.2025.f2-1 Figure 2-1 Brain activation induced by everyday objects in different > identical condition. Download Figure 2-1, TIF file.

10.1523/ENEURO.0002-25.2025.f2-2 Figure 2-2 Brain activation induced by everyday objects in different > mirror condition. Download Figure 2-2, TIF file.

Subsequently, we compared brain activation induced by everyday objects presented in the mirror versus identical conditions (mirror cost) and in the opposite direction (identical vs mirror conditions). We did not find any significant effects for either comparison, even using an exploratory cluster-wise threshold of *p* < 0.01, with a voxel-wise threshold of *p* = 0.01 (uncorrected).

Finally, we compared everyday objects presented in different versus mirror conditions (mirror priming). Similar to identity priming, we observed activation difference in a large portion of the bilateral fronto-parieto-temporo-occipital network. Specifically, greater neural responses were found bilaterally in the parietal cortex, including the left postcentral gyrus (−45, −28, 50; *t* = 7.47, 586 voxels) and the right postcentral gyrus (48, −28, 53; *t* = 6.38, 134 voxels), in the frontal regions, spanning the left posterior-medial frontal (−3, −7, 56; *t* = 6.82) and the right posterior-medial frontal (6, 5, 53; *t* = 6.95), both in one cluster with 257 voxels. Furthermore, activation difference was observed in the occipitotemporal cortex, including the ventral visual stream, with activation differences in the left fusiform gyrus (−33, −55, −13; *t* = 6.45, 201 voxels) and the right fusiform gyrus (33, −43, −19; *t* = 4.19, 115 voxels; Fig. 2*B*, Extended Data Fig. 2-2). Neural response for identity and mirror priming overlapped in the frontal and parietal cortex, as well as in the left fusiform cortex (with a sub-peak at coordinates −33, −55, −19; Fig. 2*C*).

We repeated the analysis for Braille letters but did not observe any significant effects for either identity priming (different > identical) or mirror cost (mirror > identical). No significant activation difference was found for mirror priming either (different > mirror). We speculated that the presentation times for Braille letters were too long to observe the priming effect, which is known to depend on stimulus presentation time (Schacter et al., 2004; Rączy et al., 2019).

#### ROI analysis

First, we conducted repeated-measures ANOVAs (with three conditions: identical, mirror, and different) for everyday objects with beta estimates averaged across voxels within the left occipital-temporal regions as the dependent variable. This analysis was performed in various ROIs (including anatomical location of regions in the parietal, dorsal, and ventral visual network). In all anatomical regions of the left occipital-temporal cortex, we obtained a main effect of orientation (all *F*'s > 9.78; all *p* < 0.001; *η*^2^ between 0.36 and 0.59). In each anatomical ROI, pairs of everyday objects presented in the different condition induced significantly greater response relative to the identical condition (all *p* < 0.037) and to the mirror condition (all *p* < 0.003). There was no significant activation difference between the identical and mirror condition (*p* > 0.377) indicative of mirror-invariant object processing. Similar results were obtained when combining all regions into one ROI (*F*_(2,34)_ = 18.78, *p* < 0.001; *η*^2^ = 0.53; post hoc: different > identical *p* < 0.001; different > mirror *p* < 0.001, identical > mirror *p* = 1.000; Fig. 4*A*).

Next, the ROI analysis was repeated for the parietal regions. Repeated-measures ANOVAs (with three conditions: identical, mirror, and different) were performed on participants’ beta estimates averaged across voxels within the separate anatomical masks of parietal regions. The effect of orientation was significant in each ROI (all *F*'s > 14.62; all *p* < 0.001; *η*^2^ between 0.46 and 0.66). In each ROI, pairs of everyday objects presented in the different condition induced significantly greater response than those in the identical condition (all *p* < 0.001) or mirror conditions (all *p* < 0.008). No significant activation difference was found between the identical and mirror conditions (*p* = 1.00) indicative of mirror-invariant object processing. Similar results were found when combining all regions into one ROI (*F*_(2,34)_ = 28.06, *p* < 0.001; *η*^2^ = 0.62; post hoc: different > identical *p* < 0.001; different > mirror *p* < 0.001, mirror > identical, *p* = 1.000; Fig. 4*B*).

### Experiment 2—Braille letters

In Experiment 2, the presentation times were optimized for Braille letters, and only Braille letters were presented to the participants. The longer presentation times of Braille letters in Experiment 1 were dictated by the design of the conveyor belt (Extended Data Fig. 1-1*A–D*) used to present the tactile stimuli. Hence, in Experiment 2, we optimized the stimulus presentation time for Braille letters. This time, we used an fMRI-compatible Braille device, which allowed us to present the stimuli for 800 ms—a duration shown to be optimal for such tasks (Rączy et al., 2019). Given the tactile nature of Braille reading, we aimed to strike an optimal balance between allowing participants sufficient time to accurately perceive and identify each character, while ensuring that the stimulus duration remained brief enough to preserve the temporal sensitivity required to elicit repetition suppression effects.

#### Whole-brain analysis

**Figure 3.**
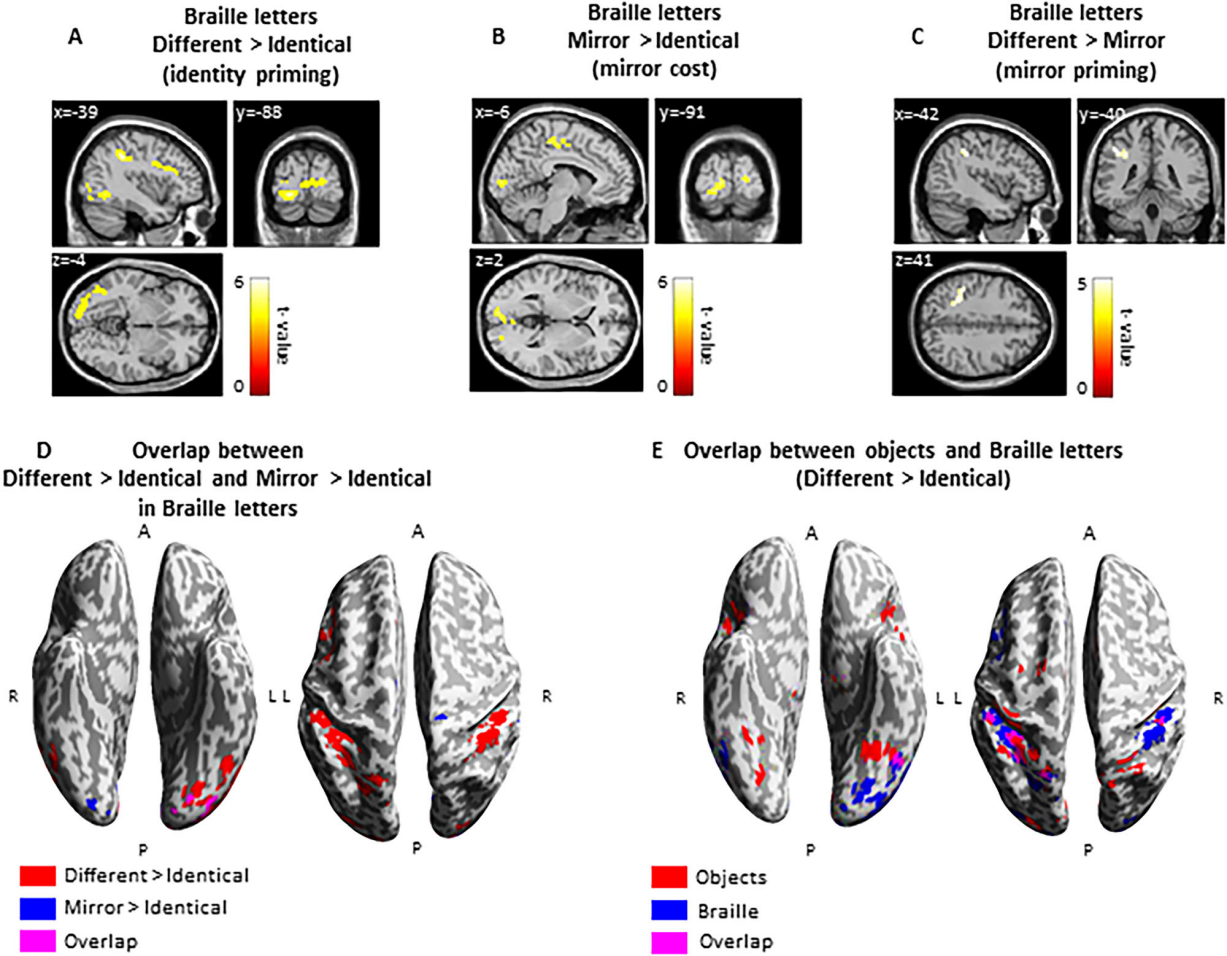
FMRI priming effects for Braille letters. ***A***, Identity priming: Braille letters induced bilateral activation in the brain’s dorsal parietal and posterior medial frontal regions. Additionally, activation was observed in the left occipito-temporal regions, particularly in the left inferior occipital gyrus, left lingual gyrus, and left middle occipital gyrus. ***B***, Mirror priming: Braille letters presented in mirror orientation induced greater activation in bilateral regions of the occipital cortex, specifically in the left calcarine and right lingual gyrus. ***C***, Absence of mirror priming: Braille letters did not produce mirror priming in occipito-temporal regions of the brain. Only the left inferior parietal lobule was activated. ***D***, Overlap between contrasts: Different > identical and mirror > identical in Braille letters revealed activation in the left inferior occipital gyrus (−21, −88, −7), illustrating how the brain distinguishes between two letters. ***E***, Activation comparison: Pairs of different Braille letters and objects evoked greater activation than pairs of the same letters and objects in the left inferior occipital gyrus (−48, −61, −13) and parietal cortex. Thresholds: ***A–D***, *p* < 0.001 unc. voxel-wise, *p* < 0.05 FWE cluster-wise; ***E***, only objects: *p* < 0.05 FWE voxel-wise, *p* < 0.05 FWE cluster-wise; R, right; L, left; A, anterior; P, posterior. For additional information, see Extended Data Figure 3-1.

First, we contrasted Braille letters presented in different > identical conditions (identity priming). This contrast showed activation difference in the parietal cortex bilaterally [left: inferior parietal lobule (−42, −40, 4; *t* = 6.00, 372 voxels); right: inferior parietal lobule (45, −37, 47; *t* = 5.49, 158 voxels)], precentral gyrus (−42, 5, 29; *t* = 3.86, 128 voxels), and in the left inferior occipital gyrus (−42, −64, −7; *t* = 4.53, 70 voxels), as well as bilaterally in the occipital pole (left: −30, −82, −13, *t* = 5.66; right: 12, −91, 8, *t* = 4.41; 314 voxels; Fig. 3*A*, Extended Data Fig. 3-1).

10.1523/ENEURO.0002-25.2025.f3-1 Figure 3-1 Brain activation induced by Braille letters in three conditions: different > identical, mirror > identical, different > mirror. Download Figure 3-1, TIF file.

Next, we contrasted Braille letters presented in mirror > identical conditions (mirror cost), for which we expected to find similar priming effects to those observed for the different > identical pairs. We found significant activation difference in the right parietal cortex (postcentral gyrus: 12, −24, 62; *t* = 5.11, 94 voxels) and bilaterally in the occipital cortex [left: inferior occipital gyrus (21, −88, −7; *t* = 4.55, 123 voxels); right: superior occipital gyrus (21, −88, 5; *t* = 4.12, 49 voxels); Fig. 3*B*, Extended Data Fig. 3-1).

Finally, we contrasted Braille letters presented in different > mirror conditions (mirror priming), and we observed a significant activation difference in the left parietal lobule (−42, −40, 41; *t* = 4.31, 57 voxels; Fig. 3*C*, Extended Data Fig. 3-1).

Additionally, we found that effects for identity priming and mirror cost overlapped in the left inferior occipital gyrus (with the sub-peak: −21, −88, −7; Fig. 3*D*). Next, we overlapped the main results of identity priming (different > identical) obtained for everyday objects and those for Braille letters, and we observed that both stimuli evoked greater activation difference in the left inferior occipital gyrus (with the sub-peak: −48, −61, −13; Fig. 3*E*).

#### ROI analysis

**Figure 4.**
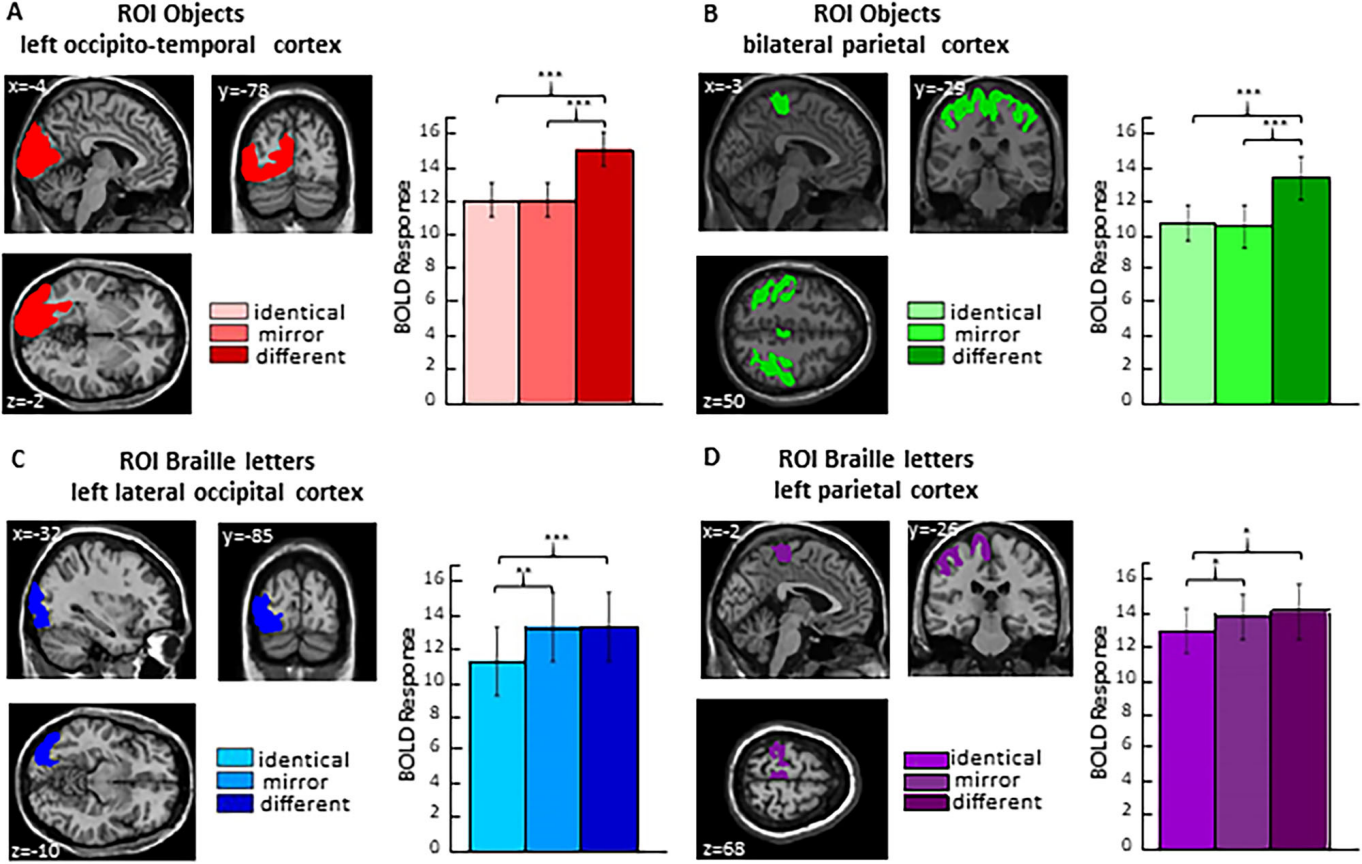
ROI results. ***A***, The analysis was performed in six anatomical ROIs within the left occipito-temporal regions for the signal change induced by three conditions (identical, mirror, and different) for objects in the first fMRI experiment. ROIs included primary visual cortex (V1 and V2), middle-temporal cortex (V5), ventral and dorsal extrastriate cortex, fusiform gyrus, and lateral occipital cortex. In all ROIs, pairs of different objects induced significantly greater activation than pairs of same and mirror objects. ***B***, The same activation patterns were observed in all ROIs in the bilateral parietal cortex. ROIs included the intraparietal sulcus, motor cortex, and primary somatosensory cortex. Both results ***A*** and ***B*** suggest that mirror invariance for everyday objects in congenitally blind individuals engages the brain in occipito-temporo-parietal regions. ***C***, We observed mirror discrimination for single Braille letters in the left lateral occipital cortex but not in the left ventral occipito-temporal cortex. ***D***, We observed mirror discrimination for single Braille letters in the left parietal cortex. Threshold levels: *p < 0.05, **p < 0.01, ***p < 0.001. Error bars represent SEM. For additional information, see Extended Data Figs. 4-1–4-8.

First, similar to the ROI analysis for the everyday objects, we conducted repeated-measures ANOVAs (with three conditions: identical, mirror, and different) for Braille letters with participants’ beta estimates averaged across all voxels within the anatomical masks as the dependent variable. Only in the left lateral occipital cortex, we found a significant effect of condition (*F*_(2,34)_ = 10.22, *p* < 0.001; *η*^2^ = 0.38), with the pattern of results characteristic for breaking mirror invariance; that is, we observed a significant difference between Braille letters presented in identical relative to mirror (mirror > identical *p* = 0.009) and different condition (different > identical *p* < 0.001) with no differences observed between different and mirror condition (Fig. 4*C*). In the primary visual cortex (V1) and middle temporal cortex, we observed a significant effect of condition (all *F*'s > 3.802; all *p* < 0.032; *η*^2^ between 0.18 and 0.26), but it did not follow the pattern of activation differences where the identical condition was significantly different from both mirror and different conditions. In the remaining ROIs of the ventral visual stream, we did not observe a significant effect of orientation either in the fusiform gyrus, VWFA, primary visual cortex (V1), or dorsal extrastriate cortex (all *F*’s < 3.18; all *p* > 0.054; *η*^2^ between 0.07 and 0.16). In the parietal cortex, we found that the effect of orientation was significant only in the left parietal cortex (*F*_(2,34)_ = 4.46, *p* = 0.019; *η*^2^ = 0.21), with significant differences observed between Braille letters presented in identical relative to mirror (mirror > identical *p* = 0.033) and different conditions (different > identical *p* < 0.042; Fig. 4*D*)[Fig eN-NWR-0002-25F5].

10.1523/ENEURO.0002-25.2025.f4-1 Figure 4-1 (A) Anatomical masks for the left occipital-temporal were created using the SPM Anatomy Toolbox 2.2b (Eickhoff et al., 2005). The left occipital-temporal regions encompassed areas such as 1) the primary visual cortex (V1 and V2 i.e., BA 17 and BA 18), 2) middle-temporal cortex (hOC5 (V5 / MT+))(, 3) ventral (V3v / V4) and 4) dorsal extrastriate cortex (hOC3d / hOC4d), 5) fusiform gyrus (Areas FG1, FG2, FG3 and FG4), and 6) lateral occipital cortex (extrastriate areas hOc4la and hOc4lp). (B) Anatomical masks for the bilateral parietal cortex created using the SPM Anatomy Toolbox 2.2b (Eickhoff et al., 2005) included: 1) the intraparietal sulcus(Areas hIP1, hIP2, hIP3), 2) motor cortex (Areas 4a and 4p), and 3) primary somatosensory cortex (Areas 1, 2, 3a, 3b). Download Figure 4-1, TIF file.

10.1523/ENEURO.0002-25.2025.f4-2 Figure 4-2 (A-H) Anatomical masks for the left occipital-temporal created using the SPM Anatomy Toolbox 2.2b (Eickhoff et al., 2005). The left occipital-temporal regions encompassed areas such as 1) the primary visual cortex (V1 and V2, i.e., BA 17 and BA 18), 2) the middle-temporal cortex (hOC5 (V5 / MT+))(, 3) ventral (V3v / V4) and 4) dorsal extrastriate cortex (hOC3d / hOC4d). All figures present individual results in individual masks for objects. Thresholds levels: *p < 0.05, ** p < 0.01, ***p < 0.001. Error bars represent S.E.M. Download Figure 4-2, TIF file.

10.1523/ENEURO.0002-25.2025.f4-3 Figure 4-3 (I-M) Anatomical masks for the left occipital-temporal created using the SPM Anatomy Toolbox 2.2b (Eickhoff et al., 2005). The left occipital-temporal regions encompassed areas such as 1) the primary visual cortex (V1 and V2, i.e., BA 17 and BA 18), 2) the middle-temporal cortex (hOC5 (V5 / MT+))(, 3) ventral (V3v / V4) and 4) dorsal extrastriate cortex (hOC3d / hOC4d). All figures present individual results in individual masks for objects. Thresholds levels: *p < 0.05, ** p < 0.01, ***p < 0.001. Error bars represent S.E.M. Download Figure 4-3, TIF file.

10.1523/ENEURO.0002-25.2025.f4-4 Figure 4-4 (A-H) Anatomical masks for the bilateral parietal cortex created using the SPM Anatomy Toolbox 2.2b (Eickhoff et al., 2005) included: 1) the intraparietal sulcus(Areas hIP1, hIP2, hIP3), 2) motor cortex (Areas 4a and 4p), and 3) primary somatosensory cortex (Areas 1, 2, 3a, 3b). All figures present individual results in individual masks for objects. Thresholds levels: *p < 0.05, ** p < 0.01, ***p < 0.001. Error bars represent S.E.M. Download Figure 4-4, TIF file.

**Figure 5. eN-NWR-0002-25F5:**
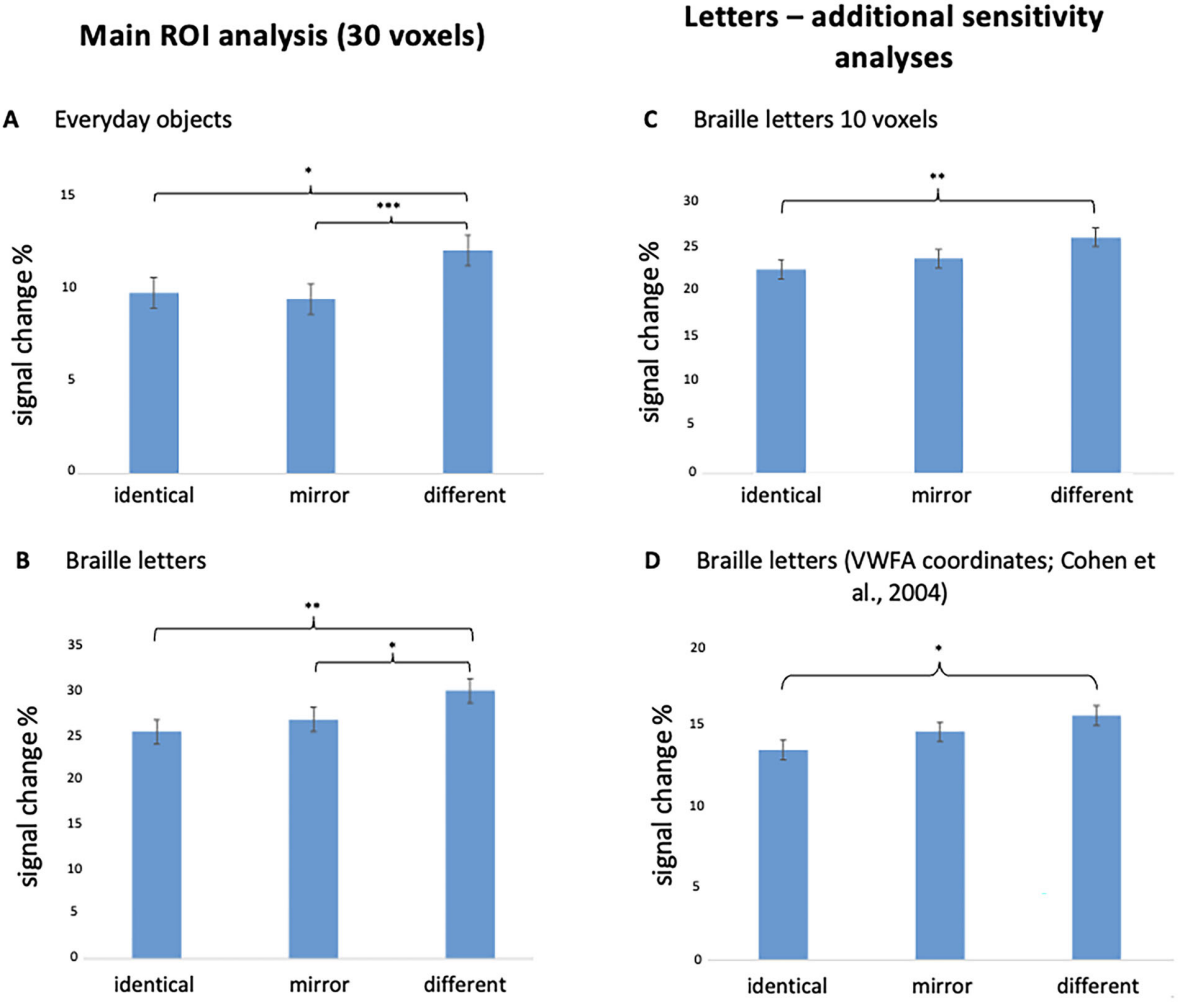
ROI Analysis for the Visual Word Form Area (VWFA). Following Rączy et al. (2019), we defined the ROI for each participant as follows: ***A***, ***B***, 30 noncontiguous voxels with the highest *t*-value in the Braille letters versus rest contrast. ***C***, 10 noncontiguous voxels with the highest *t*-value in the Braille letters versus rest contrast. In this case, activation in two participants did not reach 10 voxels, so they were excluded. In ***A–C***, ROIs were defined within the anatomically defined boundaries of the given region (Zmax = −10, Zmin = −25, Ymax = −70, Ymin = −40, Xmax = −60, Xmin = −30). In ***D***, the ROI was defined using the MarsBaR toolbox as an 8 mm radius sphere with the canonical VWFA coordinates reported by Cohen et al. (2004; MNI: *x* = −41, *y* = −57, *z* = −16). ***A***, For objects, a main effect of orientation was observed (*F* = 8.40, *p* = 0.001, *η*^2^ = 0.33). Pairs of everyday objects presented in the different condition induced significantly greater activation than those in the identical condition (*p* = 0.023) and the mirror condition (*p* = 0.002). There was no significant difference between the identical and mirror conditions (*p* = 1.000). ***B***, For letters based on 30 noncontiguous voxels, we found a main effect of orientation (*F* = 7.78, *p* = 0.002, *η*^2^ = 0.34). Pairs of Braille letters presented in the different condition induced significantly greater activation than those in the identical condition (*p* < 0.001) and the mirror condition (*p* = 0.031). There was no significant difference between the identical and mirror conditions (*p* = 0.300). ***C***, For letters based on 10 noncontiguous voxels, we found a main effect of orientation (*F* = 7.78, *p* = 0.002, *η*^2^ = 0.34). Pairs of Braille letters presented in the different condition induced significantly greater activation than those in the identical condition (*p* = 0.009) but did not reach significance compared with the mirror condition (*p* = 0.138). There was no significant difference between the identical and mirror conditions (*p* = 0.186). ***D***, For letters, based on an 8 mm radius sphere centered on the canonical coordinates reported by Cohen et al. (2004; MNI: *x* = −41, *y* = −57, *z* = −16), we found a main effect of orientation (*F* = 43.25, *p* < 0.001). Pairs of Braille letters in the different condition elicited significantly greater activation than those in the identical condition (*p* = 0.019) but did not differ significantly from the mirror condition (*p* = 0.514). No significant difference was observed between the identical and mirror conditions (*p* = 0.413). Threshold levels: *p < 0.05, **p < 0.01, ***p < 0.001. Error bars represent SEM.

10.1523/ENEURO.0002-25.2025.f4-5 Figure 4-5 (A-H) Anatomical masks for the left occipital-temporal were created using the SPM Anatomy Toolbox 2.2b (Eickhoff et al., 2005). The left occipital-temporal regions encompassed areas such as 1) the primary visual cortex (V1 and V2, i.e., BA 17 and BA 18), 2) the middle-temporal cortex (hOC5 (V5 / MT+))(, 3) ventral (V3v / V4) and 4) dorsal extrastriate cortex (hOC3d / hOC4d). All figures present individual results in individual masks for Braille letters. Thresholds levels: *p < 0.05, ** p < 0.01, ***p < 0.001. Error bars represent S.E.M. Download Figure 4-5, TIF file.

10.1523/ENEURO.0002-25.2025.f4-6 Figure 4-6 (I-M) Anatomical masks for the left occipital-temporal were created using the SPM Anatomy Toolbox 2.2b (Eickhoff et al., 2005). The left occipital-temporal regions encompassed areas such as 1) the primary visual cortex (V1 and V2, i.e., BA 17 and BA 18), 2) the middle-temporal cortex (hOC5 (V5 / MT+))(, 3) ventral (V3v / V4) and 4) dorsal extrastriate cortex (hOC3d / hOC4d). All figures present individual results in individual masks for Braille letters. Thresholds levels: *p < 0.05, ** p < 0.01, ***p < 0.001. Error bars represent S.E.M. Download Figure 4-6, TIF file.

10.1523/ENEURO.0002-25.2025.f4-7 Figure 4-7 (A-H) Anatomical masks for the bilateral parietal cortex created using the SPM Anatomy Toolbox 2.2b (Eickhoff et al., 2005) included: 1) the intraparietal sulcus(Areas hIP1, hIP2, hIP3), 2) motor cortex (Areas 4a and 4p), and 3) primary somatosensory cortex (Areas 1, 2, 3a, 3b). All figures present individual results in individual masks for Braille letters. Thresholds levels: *p < 0.05, ** p < 0.01, ***p < 0.001. Error bars represent S.E.M. Download Figure 4-7, TIF file.

10.1523/ENEURO.0002-25.2025.f4-8 Figure 4-8 Localizer –Experiment 2. The statistical map obtained in the localizer scan. Reading Braille letters activated the typical reading network of the sighted. Thresholds: (A) p < 0.001 unc. voxel-wise, p < 0.05 FWE cluster-wise; Download Figure 4-8, TIF file.

#### VWFA ROI

For everyday objects, a main effect of orientation was observed (*F* = 8.40, *p* = 0.001, *η*^2^ = 0.33). Pairs of everyday objects presented in the different condition induced a significantly stronger response than those in the identical condition (*p* = 0.023) and the mirror condition (*p* = 0.002). There was no significant difference between the identical and mirror conditions (*p* = 1.000; Fig. 5*A*).

For Braille letters (ROI based on 30 noncontiguous voxels), we found a main effect of orientation (*F* = 7.78, *p* =0.002, *η*^2^ = 0.34). Pairs of Braille letters presented in the different condition induced a significantly stronger response than those in the identical condition (*p* < 0.001) and the mirror condition (*p* = 0.031). There was no significant difference between the identical and mirror conditions (*p* = 0.300; Fig. 5*B*). When we restricted the ROI to 10 noncontiguous voxels when defining it, we found a main effect of orientation (*F* = 7.78, *p* = 0.002, *η*^2^ = 0.34). Pairs of Braille letters presented in the different condition induced a significantly stronger response than those in the identical condition (*p* = 0.009) but did not reach significance compared with the mirror condition (*p* = 0.138). There was no significant difference between the identical and mirror conditions (*p* = 0.186; Fig. 5*C*). Finally, for the VWFA defined as an 8 mm radius sphere centered on the canonical coordinates reported by Cohen et al. (2004; MNI: *x* = −41, *y* = −57, *z* = −16), we found a main effect of orientation (*F* = 43.25, *p* < 0.001). Pairs of Braille letters in the different condition elicited significantly greater activation than those in the identical condition (*p* = 0.019) but did not differ significantly from the mirror condition (*p* = 0.514). No significant difference was observed between the identical and mirror conditions either (*p* = 0.413; Fig. 5*D*).

Overall, either in the whole-brain analysis or in multiple ROI analyses with various definitions of VWFA, we did not observe the neural signature for breaking of mirror invariance for letters in this region; that is, neural response in mirror condition did not differ from the condition in which two letters shared the same shape and orientation.

## Discussion

The present study investigated whether the mirror invariance phenomenon can be observed for tactile objects in congenitally blind individuals and whether it is specific to regions previously reported for mirror-invariant visual object recognition. Secondly, we aimed to determine the locus of repetition suppression for identical—but not mirror—Braille letters, a neural signature of breaking mirror invariance, in congenitally blind individuals and whether it overlapped with the location of the sighted Visual Word Form Area (VWFA).

We obtained two main findings. First, we identified brain regions that in congenitally blind individuals processed tactile objects in a mirror-invariant way. Repetition suppression for identical and mirror pairs of everyday objects was observed in parietal and occipital regions, specifically in the lateral occipital complex (LOC) and anterior parts of the ventral visual stream. Second, the left parietal regions and the LOC—but not the anterior ventral occipital temporal cortex (vOTC; the sighted VWFA)—exhibited repetition suppression for identical but not mirror Braille letters, a key signature of reading expertise found in the sighted.

Mirror invariance has, so far, only been reported for visual objects (Dilks et al., 2011; Pegado et al., 2011). The repetition suppression found for identical and mirror pairs of everyday objects in the occipitotemporal and parietal cortex of blind individuals—overlapping with regions involved in mirror-invariant visual object recognition in sighted individuals—extends these findings, demonstrating that this perceptual bias is not exclusive to the visual modality (Rollenhagen and Olson, 2000; Xu et al., 2023). These results align with Hannagan et al.’s (2015) proposal that neurons, particularly in higher-level visual regions, are specialized for specific computations—such as object recognition or geometry processing—rather than being tied to a particular sensory modality, in line with the metamodal organization of the brain (Pascual-Leone and Hamilton, 2001).

The parieto-occipital regions of sighted individuals are engaged in visual (Dehaene et al., 2010; Dilks et al., 2011) and tactile object recognition (Kitada et al., 2014; Fujimoto et al., 2017). In fact, parietal regions have been linked to visual and haptic object manipulation and to be particularly crucial for the control of spatially guided actions (Lucan et al., 2010; Kim and Zatorre, 2011). The latter has been suggested to result from the anatomical proximity of parietal regions to the sensorimotor and occipital cortices. In line with this notion, high functional connectivity (FC) between these regions has been repeatedly reported in sighted individuals (Rolls et al., 2023). An even higher FC between parietal and occipital regions than in the sighted group has been reported in blind individuals, which has been attributed to their overtrained tactile skills (Collignon et al., 2013; Qin et al., 2015; Jiao et al., 2023). Parietal and occipital areas are often simultaneously activated in blind individuals during tactile (Stilla et al., 2008) and spatial imagery (Vanlierde et al., 2003) and spatial working memory (Bonino et al., 2008) tasks. The posterior parietal cortex (PPC) has been suggested to integrate spatial features of an object into a multimodal high-level representation (Rolls et al., 2023), while the inferolateral occipitotemporal cortex has been found to be activated during shape discrimination (Xu et al., 2023), independent of input modality—as long as the geometric information is conveyed (Hannagan et al., 2015). In fact, Xu and colleagues (2023) have demonstrated that while the parieto-occipital cortices of both blind and sighted individuals were activated during the auditory processing of shape and category of man-made objects, the inferolateral occipitotemporal cortex showed higher activation in shape verification task than in a conceptual task (in which participants judged the conceptual similarity of objects), corroborating that vOTC neurons are “apt at recognizing the shapes of objects” (Hannagan et al., 2015, p. 379) independent of modality. Two recent behavioral studies with blind individuals (de Heering et al., 2018; Korczyk et al., 2024), one involving participants overlapping with the current study (Korczyk et al., 2024), demonstrated that blind individuals “break” mirror invariance for Braille letters—that is, they exhibit high expertise in letter-orientation discrimination and, consequently, high reading proficiency—similar to sighted participants. In fact, De Heering et al. (2018) were able to demonstrate that in blind individuals, mirror-image discrimination generalizes to other scripts (e.g., Latin letters) and object categories, such that the more a stimulus resembles a letter, the more difficult it becomes to judge the shape of two mirror objects as identical.

To extend these findings to familiar stimuli, we conducted a behavioral study with largely the same sample as in the current fMRI experiment (Korczyk et al., 2024). While De Heering et al. (2018) used abstract shapes such as polynomial chains, in Korczyk et al. (2024)participants were presented with familiar geometric forms and 3D everyday objects—the latter being identical to those used in the present neuroimaging study. In the congenitally blind group, Korczyk et al. (2024) observed the smallest mirror drop for linguistic stimuli (Braille letters), with a linear increase in mirror drop as stimulus similarity to letter shapes decreased. Notably, the highest mirror drop was observed for 3D objects, suggesting that these were least susceptible to mirror contrast effects and thus most likely processed in a mirror-invariant manner (for the further discussion, see Korczyk et al., 2024). These behavioral findings align with the current fMRI results of repetition suppression for both identical and mirror-pairs of tactile objects and are consistent with previous findings in the visual modality for nonlinguistic objects as well (e.g. planes, flags etc.; Pegado et al., 2011).

FMRI studies in sighted readers consistently demonstrated that the VWFA responds to both objects and words (Dehaene and Cohen, 2007; Szwed et al., 2011), reflecting the fact that letters constitute a subclass of visual objects (see neural recycling hypothesis; Dehaene and Cohen, 2007). For fluent reading, however, typical mirror-invariant processing of the vOTC must be suppressed: In expert readers repetition suppression occurs for both identical and mirrored object pairs, but only for identical, and not mirrored, letter pairs as compared with different stimulus pairs (Pegado et al., 2011). Within this framework, we hypothesized that if computations in the anterior vOTC (sighted VWFA) are modality-independent and parallel those of sighted readers, mirror-invariant responses would be present for tactile objects but not for Braille letters in this region in congenitally blind individuals. Contrary to this prediction, in the current study repetition suppression for identical—but not mirror—Braille letters was found in the broad areas of the parietal cortex and the LOC, but it was notably absent in the anterior vOTC (VWFA) of congenitally blind individuals. This indicates significant differences in reading-related orthographic processes between sighted and blind individuals. Specifically, in blind individuals reading network seems much more broadly distributed than the localized VWFA processing found in the sighted group (Pegado et al., 2011). Nevertheless, convergence can be observed: Regions beyond the VWFA seem to play a significant role in visual reading too. For instance, the posterior parietal cortex (PPC), considered as an attentional area, has been linked to grapheme–phoneme mapping, letter-identity processing (Reilhac et al., 2013), and visual working memory, particularly when reading becomes more attentionally demanding—e.g., when word forms are degraded or during spelling-like reading tasks (Deschamps et al., 2014). In blind individuals, the PPC appears to be the primary locus of reading-related orthographic processing: Responses to Braille words in these regions were lateralized according to language processing areas but not to the reading hand (Tian et al., 2023), and PPC activity increased with the number of letters in the uncontracted word version (Liu et al., 2023), suggesting its role in letter identification and letter retrieval. Our finding of repetition suppression for only identical Braille letters in the PPC of blind individuals corroborates these results and suggests that this region—possibly initially specialized for attentional processing—may have taken on an expanded role in tactile reading, likely facilitated by its proximity to the somatosensory cortex. This role may include involvement in an intermediate stage of letter-identity processing.

The observed priming effects for letters were specific to the LOC region as well. Here, it could be speculated that the LOC serves as the primary region for breaking mirror invariance of Braille letters and hence, engages in grapheme-to-phoneme mapping in tactile reading (Brem et al., 2010; Pegado et al., 2014), similar to the sighted VWFA (Pegado et al., 2014). Hannagan et al. (2015) proposed that the primary computational role of the LOC is the construction of 3D models of perceived objects. This assumption was based on studies demonstrating LOC activation in object recognition tasks involving objects presented in visual and tactile modalities—both of which inherently convey geometric information—and for sounds but only when auditory input was engineered to carry shape-related cues (Amedi et al., 2007). Braille characters are small-scale 3D objects that require fine-grained spatial processing. Thus, they likely possess a “tactile-object component” in addition to being orthographic units. We thus hypothesize that the LOC serves as an early processing stage in Braille reading, where orientation-specific features are extracted, and initial grapheme-to-phoneme conversion occurs. Next the letters are presumably integrated into bigger orthographic units. In fact, the LOC has been reported to be sensitive to the linguistic properties of visual Braille and Latin words in sighted (visual) Braille readers (Cerpelloni et al., 2024) and to feature a similar activity pattern to their VWFA. Finally, an MEG study in sighted readers indicated the presence of a Letter Form Area in the LOC, where single letters are encoded before more complex letter combinations, such as bigrams (Thesen et al., 2012; see Zhan et al., 2023 for fMRI results).

In sighted literate individuals, the neural signature of breaking mirror invariance has been associated with the VWFA (Dehaene et al., 2010; Pegado et al., 2011). The VWFA emerges in regions dedicated to object recognition, as letters from many visual scripts are composed of line junctions that are apparent in the visual scene (McCandliss et al., 2003; Price and Devlin, 2011). Recent studies in sighted Braille readers have, however, found selective VWFA activation in response to both tactile and visual Braille alphabets, which lack these visual characteristics (Siuda-Krzywicka et al., 2016; Cerpelloni et al., 2024). Moreover, Striem-Amit et al. (2012) found letter-selective activation in blind individuals—in a location overlapping with the sighted VWFA—while presenting blind individuals with three-letter consonant strings via sensory substitution device. Finally, Rączy et al. (2019) found, in congenitally blind individuals, repetition suppression for identical but not different pairs of tactile—and not auditory—pseudowords in the anterior vOTC (sighted VWFA). These findings suggested that, at least to some extent, the anterior vOTC of blind individuals preserves its underlying computation—orthographic processing.

Results of the current study significantly extend these previous findings and draw a different, more complete picture, one in which a hallmark component of reading—“breaking” of mirror invariance for letters—in blind individuals is carried out by considerably broader and distinct brain regions, including the parietal cortex and broad swatches of the LOC, but not the VWFA. These data suggest that blindness leads to a reorganization of the reading network: New, noncanonical regions—likely leveraging their intrinsic computational predispositions (e.g., geometric processing)—appear to expand their functional roles to novel tasks such as letter-identity recognition. At the same time, classical reading regions identified in sighted individuals, such as the anterior VOTC (VWFA), following blindness retain only part of their typical orthographic functions, such as the analysis of bigrams and words (Reich et al., 2011; Striem-Amit et al., 2012; Rączy et al., 2019). Notably, unlike in sighted individuals, these regions also extend their role to higher-level cognitive processing (Bedny, 2017), which is likely supported by enhanced FC between the visual cortex and higher-order cognitive networks that convey rich semantic and language-specific information to the visual cortex (Bedny, 2017; Saccone et al., 2024). In fact, the latter has been lately corroborated by findings that the macroscale organization of the visual cortex following blindness is radically altered, with a markedly different structure-function coupling in visual areas (Koba et al., 2025).

Finally, neither identity nor mirror priming effects were found in somatosensory regions. Activation in these regions was observed only in the different > mirror contrast, the only condition in which Braille letters differed in the number of dots, further supporting the role of somatosensory cortex in low-level sensory processing (Liu et al., 2023).

Several limitations should be acknowledged in this study. We used pairs of Braille letters and everyday objects mirrored along the vertical axis. To thoroughly investigate the mirror invariance phenomenon following congenital visual deprivation, future studies should also incorporate mirror stimuli along the horizontal axis (De Heering et al., 2018). To assess the impact of object familiarity (Margalit et al., 2016), a broader range of stimuli, such as geometric figures and polynomial chains (De Heering et al., 2018), should be employed. Furthermore, investigating the role of context and top-down modulation from higher-tier areas in breaking mirror invariance for Braille letters would be of interest. Lastly, we acknowledge the absence of a sighted control group; however, including such a group would have introduced critical limitations. First, embossed letters are not typically used for tactile reading by either blind or sighted individuals, making them an ecologically invalid and unfamiliar stimulus. Second, sighted individuals cannot read tactile Braille without extensive training. Even after a 9 month course (Bola et al., 2016), their proficiency remains at a second-grade level. While proficient blind Braille readers achieve an average reading speed of ∼60 words per minute (Rączy et al., 2019), sighted Braille readers reach only approximately five words per minute (Bola et al., 2016, Rączy et al., 2020) and decoding even a single Braille letter would require considerably more time in this group. In light of this, and given the findings of Experiment 1 of the current study (narrow temporal window for priming to occur) even if we could find sighted tactile Braille readers, introducing this group would likely introduce confounds, limiting the interpretability of the findings.

In summary, our study identified brain regions involved in the mirror-invariant processing of haptic objects in blind individuals. Among these regions, the parietal and lateral occipital cortices exhibited neural patterns indicative of breaking mirror invariance for Braille letters. While these regions are part of a coactivated network engaged in tactile object recognition in blind individuals, we hypothesize that they serve distinct functions. The parietal regions likely process letter features such as orientation and spatial relation between two objects, serving as an initial step in distinguishing mirror and non-mirror letters. In contrast, we propose that the LOC integrates shape information with letter-identity representation, possibly supporting the grapheme-to-phoneme conversion. Together, these results suggest that reading-related orthographic processes in blind individuals are significantly different than in sighted humans, resulting in a reading network reorganization in the blind group. Strikingly, the strong specialization for orthographic processing, in particular the “breaking” of mirror invariance for letters, found in the visual ventral stream of the sighted was notably absent in blind individuals suggesting that reading can be supported by different configurations of brain areas. The ventral visual stream may thus not be universally specialized for a specific task (such as reading), but rather for performing certain computations—which, following congenital visual deprivation, can be repurposed.

## Data Availability

Data will be made available upon reasonable request to the coresponding author.

## Synthesis

Reviewing Editor: Frederike Beyer, Queen Mary University of London Decisions are customarily a result of the Reviewing Editor and the peer reviewers coming together and discussing their recommendations until a consensus is reached. When revisions are invited, a fact-based synthesis statement explaining their decision and outlining what is needed to prepare a revision will be listed below. The following reviewer(s) agreed to reveal their identity: Tania Fernandes, Jacek Matuszewski. Note: If this manuscript was transferred from JNeurosci and a decision was made to accept the manuscript without peer review, a synthesis may not be available.

Both reviewers highlight the novelty of your reserach and its interest to the field. As you can see in their comments below, they appreciate the challenges of this type of work and the quality of your reserach design. They also highlight important areas for improvement, in particular clarifying some of the terminology used, addressing issues around interpretation of null findings, and expanding the discussion of relevant previous research. Please see their detailed comments below.

Reviewer 1

I read this manuscript (ms) with interest, as the investigation of mirror-image processing and the impact of literacy acquisition on it provides a valuable perspective on the cognitive interplay between object recognition and reading - which has typically been studied in the visual modality. This interaction remains poorly understood, particularly in other sensory modalities such as touch, making this study both timely and relevant. The present research is original, well-designed, and innovative, with strong potential to contribute to this field.

Although I was not involved in the initial round of evaluation, I have consulted the previous reviewers' comments after reading the ms. The authors have done a very good job revising their work. Likely due to this being a revised version, I have only three major comments that require the authors' attention. Addressing these points will hopefully improve the manuscript and enhance the clarity of its potential contribution to the field.

1) Precision in the terminology adopted will assist in clarity.

Specifically:

1.1. The term "priming" usually refers to the comparison of a condition on which a prime has a particular relation with a target (either it is the same as or shares some property with the target) relative to a control, baseline condition on which prime and target have no relation. Therefore, it is not correct to refer to mirror-image discrimination as "mirror priming" (as the authors do on pp. 12-13, lines 263-282, and p. 14, lines 320-325) because this latter nomenclature should be used when a mirrored condition (mirrored orientation and same identity; e.g., d and b) is compared to an UNRELATED condition (different orientation and different identity: e.g., p and z) and not to an identity condition (same orientation and same identity: e.g., d and d). The control, baseline condition is very important, and hence, when referring to "priming" (or to any other contrast) please be explicit on which baseline condition is being considered (e.g., p. 3, lines 54-60).

When mirrored and identity conditions are compared to one another, then what is being examined is whether there is a difference in one relative to the other. This would reflect mirror-image discrimination (or a mirror cost) and NOT mirror invariance, unless the difference between them was null (which was the case in the present study, as reported on pp. 12-13). Therefore, in this latter case, this result is problematic, given the null-hypothesis significance testing, and hence, it must be further confirmed by the observation of an advantage of similar magnitude for (i) the identity condition relative to a control condition and for (ii) the mirrored condition relative to a control condition. Only the latter contrast (contrast ii) could be referred to as "mirror priming".

1.2. "the locus of letter shape processing" (p. 4, lines 83-91). The rationale for arguing that a neural locus on which mirror priming is found for non-letter objects and mirror discrimination (identity priming but no mirror priming effect) is found for letters is "the locus of letter shape processing" needs to be improved. Mirrored letters have the same shape (either in geometrical terms in the visual modality or as a matrix or pattern in Braille); the only difference between mirrored letters (in either modality) is in orientation and not in shape. Thus, orthographic processing is not about shape processing. To be clear, my concern here is the reference to "shape" when referring to letter processing.

2. Method and results of Experiment 2. The authors indicate that they have tested all 32 letters of the Braille Polish alphabet (p. 6, line 132: how many of these letters are mirrored letters?) but that 36 pairs were presented in each condition (p. 8, line 173: were all these 36 mirrored pairs mirrored reversible letters?). If not, then in the mirrored condition, did the authors conduct separated analysis for reversible letters (that is, pairs whose mirrored versions correspond to two different real letters; e.g., d and b) and for nonreversible letters (that is, pairs on which one is a real letter, and the mirrored version is not a letter of the script)? Based on previous studies with sighted readers of the Latin alphabet (e.g., Brossette et al., 2022. https://doi.org/10.3758/s13414-021-02405-y; Fernandes et al., 2022, https://doi.org/10.1016/j.jml.2022.104375; Perea et al., 2011, https://doi.org/10.1016/j.jml.2011.04.005) aggregating the results of these two letter sets could be misleading, as mirrored versions of reversible and of nonreversible letters are processed differently during visual word recognition because only reversible letters correspond to different letter units.

3. In the Discussion's section, the discussion of previous behavioral results regarding mirror-invariance and mirror-image discrimination by congenital blind (CB) participants needs to be improved. The present study follows from previous studies, which is acknowledged by the authors along the ms, but the behavioral results from De Heering et al. (2018; De Heering &Kolinsky, 2019) must be considered in the Discussion. It is not enough to refer to this study (which the authors did on pp. 17-18, lines 395-400). It is critical to discuss the differences between that study and the present study. In particular, De Heering et al. found that, at least behaviorally, mirror-image discrimination generalized to other visual categories given that the mirror cost, that is, the disadvantage in shape-based judgments of mirrored pairs relative to identical pairs (in tactile modality), was found not only for Braille letters but also for embossed Latin-alphabet letters and geometric shapes, which in turn was negatively correlated with Braille reading fluency (the larger the mirror cost, the better the reading fluency of CB readers), whereas the present study found mirror invariance for non-letter items, and hence, this discrepancy must be discussed. I am not arguing that these findings are contradictory to the present results, as one study regards behavioral data and the present study regards BOLD-signal, but they should be discussed, especially because both studies have implications for future research. This point was already raised by another reviewer (Reviewer 1), so the authors should explicitly address this issue and improve this part of their discussion. I also think that the methodological point raised by Reviewer 2 regarding the absence of a control (sighted) group is relevant as well as the authors' answer to it. Therefore, it should also be considered in the Discussion, acknowledging this potential limitation (absence of a control group), as well as the authors' rationale for not including such a control group.

4) Minor aspects.

4.1.Abstract.

I think that it is very confusing to refer to previous results regarding mirror-image discrimination in the VWFA by sighted readers because (1) these results are not from the present study (so, to refer to them, please follow APA guidelines, presenting the explicit reference to that study); (2) the present study did not examine sighted readers (which was the population examined in that previous study, and hence, if anything such reference in the abstract should be explicit); (3) the present results with CB readers are not consistent with those former results. My suggestion would be to remove that reference from the abstract, or at least to revise this passage.

4.2. Significance Statement

For the sake of clarity and precision please do not refer to mirror invariance as a perceptual bias to recognize rotated objects, given that mirror invariance is about one particular type of orientation contrast, that is, mirror images, It does not generalize to rotations in the picture plane. Indeed, plane rotations, even when with the same 180º angular difference as mirror images (e.g., b and q; p and d) are not recognized as identical (or invariant).

4.3. Introduction.

p.3, lines 54-56: the baseline condition is missing: "(...) a decrease in neural response to a target stimulus (...) when it is preceded by an identical stimulus (referred to as a prime)" - RELATIVE TO WHAT? DECREASE IN COMPARISON TO A BASELINE, UNRELATED STIMULUS, isn't it?

4.4 Method.

p. 8, lines 179-182. In which way did the procedure adopt minimized top-down effects?

4.5. Please fixed the typos:

- abstract: "congenially blind"-> congenital blind

- p. 4, lines 83 and 84: mirror-priming -> mirror priming

- p. 4, line 84: identical -> identity

- p. 9, line 194: "we can only measure accuracy" -> "we only measured accuracy

- p. 15, line 347: "primary visual cortex (V2)" -> "primary visual cortex (V1)".

Reviewer 2

In their manuscript, Korczyk et al,. investigate how congenitally blind individuals break mirror invariance for objects and tactile Braille letters with two partially-dependent fMRI studies. I find the study interesting and worth publishing. However, I feel like the analyses in their current form are slightly disconnected from the hypotheses stated in the introduction and inferences made in the discussion. Therefore, in my opinion, the manuscript (and its readers) would benefit from streamlining the results and adding analyses that explicitly test stated hypotheses and support conclusions. I am looking forward to seeing a revised version of the manuscript. Please find more points listed below.

Major Points:

1. [Introduction] Hypotheses and rationale for the study (in terms of neuronal mechanisms and using fMRI) seem lacking in the introduction. The authors focus on mirror invariance and mainly just mention VWFA in one line, but list then multiple regions from the occipital and parietal cortex and ROIs in the methods/results. I understand that the 650-word limit is not easy to follow but please include more information about the brain networks studied here.

2. [General/Methods] Maybe this is my limitation in understanding the repetition-suppression paradigms (if that is the case, I am eager to learn from the authors' response), but what exactly is the role of "different" condition and "identity priming" in this manuscript and how does it relate to the hypotheses? If I understand it correctly, authors use two contrasts (different > mirror &different > identical) and compare the similarity of the output maps, inferring that if the maps are similar then mirror pairs are represented similarly to identical pairs. If that is the case, why not run direct mirror > identical contrast instead, expecting no differences if the objects are perceived similarly (like authors do in Figure 3B).

3. [General/Discussion] How long do priming effects in repetition-suppression paradigms last? Given the fact that most of the studies are performed with visual stimuli and tactile perception is inherently slower, could that impact the results? Please add relevant information to the discussion.

4. [General/Discussion] Can tactile letters also be treated as objects that are explored and "built/rendered with 3D modelling" as the authors state in the discussion? Could this explain the partially overlapping effects between objects and letters in the LOC? In other words, could letters have their "tactile object component" in addition to their linguistic/orthographic component during perception? Would it trigger activity in LOC apart from S1? Is it possible to disentangle these effects with the control conditions used in this study (or other studies)? It would be ideal to run an analysis that tries to separate these effects empirically, but if authors find that not possible with the current design at least try to discuss that possibility in the discussion.

5. [Methods/Results] Throughout the manuscript the authors make inferences about similarities, differences, conjunctions and disjunctions between conditions and stimuli classes without explicitly testing them. I understand that it is tricky to test conjunction between objects and letters at the whole brain level given 2 separate experiments with partially overlapping subjects, but conjunctions between conditions should not be an issue. Moreover, the authors state that there is specificity to objects or letters in a given region making inferences just from the lack of overlap between whole-brain contrasts. Please include direct interaction analyses in the manuscript, at least for the code ROIs related to hypotheses (see point 6). Going back to the mixed design of repeated measures (2 experiments) for most, but not all, subjects, a linear mixed effects modelling should handle this.

6. [Methods/Results] Building upon previous point, I am confused with the semi-qualitative nature of inferences here, especially in the rationale for ROI selection. In the introduction and hypotheses authors write about distinction between LOC and VWFA ( specifically, "sighted VWFA", Lines 83-91), however such analyses is nowhere to be found in the paper. Instead, authors mix-and-match different anatomical masks from the Anatomy toolbox into one "left occipital-temporal cortex" (with individual masks displayed in the supplementary materials) - how does that relate to the hypothesis? Where exactly is it tested? It seems like an obvious thing to do to include dedicated LOC and VWFA masks - either from canonical coordinates reported by Cohen and Dehaene or from online data repositories like neurosynth) - or even author's own localizer overlapped with canonical locations? Alternatively, use a VWFA also studied in the blind tactile reading context, eg., Reich et al., 2011, Kim et al., 2017, Dzięgiel-Fivet et al., 2021, Matuszewski et al., 2025. Here, instead of specifically testing the hypothesis, authors use statements like "investigating whether it overlaps with the location of sighted VWFA" - which is a qualitative inference to say the most. Alternatively, If the authors are interested in division between anterior and posterior vOTC I would suggest to use FG2 and FG4 anatomical masks from Anatomy toolbox, which colocalize with recent divisions in vOTC in the context of reading (namely, mOTS and pOTS or "VWFA1" and "VWFA2", see Lerma-Usabiaga et al., 2018, Yablonski et al., 2024) since a lot of speculations about anterior and posterior vOTC are written in the discussion (rightfully so!).

Minor Points:

7. [Introduction] Please try to add smoother transitions between paragraphs in the introduction, the introduction seems very dissected now

8. [Introduction] Line 83 please explain "identity priming" vs "mirror priming" in previous sections at least with one sentence, this phrase appears for the first time in the hypotheses and is explained better only later in the methods/results - it would make the manuscript easier to read.

9. [Methods] Please add run times for the experiments to methods section

10. [Methods] How was the precise presentation time (1500 ms / 2000 ms) controlled in Experiment 1 if the experimenter moved the conveyor belt?

11. [Methods] How many catch trials there were in the Experiment 1 and were they discarded during the analyses? Lines 203-206 regarding regressors in the GLM suggest that they were not discarded. If so, please include them as separate regressors of no interest. Additionally, consider adding auditory cues as regressors.

12. [Methods] Lines 208-213 - it would be nice and beneficial to the reader to link these contrasts to hypotheses. Alternatively, a sentence or two could be added in the results paragraph when describing them.

13. [Methods] Lines 221-225 Consider adding more details about the localizer and including it in the task / runs specifications, not later in the ROI part.

14. [Methods] Lines 244-245 How exactly were the multiple comparisons performed? Did the authors correct the results by the number of selected ROI masks and tested conditions within each ROI? In other words, what number were the p values multiplied by?

15. [Methods] What is the reason that the authors opted to use six-dot characters as gaps between pairs of stimuli in letter presentation instead of empty spaces? Tactile perception is already more complex and sequential than visual perception, and authors aim to make indirect comparisons with visual processing in sighted cited across the literature. Could this design impact behavioural performance and increase the difficulty of the comparisons in each pair as opposed to just presenting two letters with a blank space between them?

16. Line 70 "similar" -> "similarly"

17. [Results] Be cautious not to use word "activation" in the context of discussing CONTRAST results (which compare two conditions), since it's difference between two types of activations instead of activation per se. Consider changing it to "stronger/weaker response [in condition X]" or "activation differences" (e.g., Lines 254, 315, 327)

18. [Results] I find the term "fronto-parieto-temporo-occipital network" a bit confusing and redundant given the fact that it covers all brain lobes. I would remove it and list the regions instead, unless the global range of the network is exactly what the authors want to highlight (in that case, keep it).

19. [Results] Lines 341-342 - This is confusing. In the first part of the sentence the authors state significant differences between mirror and identical conditions, and in the second part of the sentence they say there were no differences between mirror and identical conditions. Is that a typo? Did the authors mean mirror and different in the second part (Fig 4C)? Please be precise with your results description.

20. Line 129 - a six dot is technically a character ("#"), not a letter

21. Lines 247-248 please add range to these results (best and worst performers)

22. Line 320 / Figure 3B &C - In the text authors define "mirror priming" as a mirror > identical contrast, yet Figure 3C (not B) is labelled as mirror priming with a different > mirror contrast. Is that a typo on the figure label or am I missing the details of the analyses presented in Figure 3C? How is that mirror priming and how does it relate to the rest of the analyses?
