# Supplementary figures and images for: Repetition Suppression for Mirror Images of Objects and Not Braille Letters in the Ventral Visual Stream of Congenitally Blind Individuals
Source: eNeuro. 2026 Jan 9;13(1):ENEURO.0002-25.2025. doi: 10.1523/ENEURO.0002-25.2025 (PMC12834325; doi:10.1523/ENEURO.0002-25.2025)

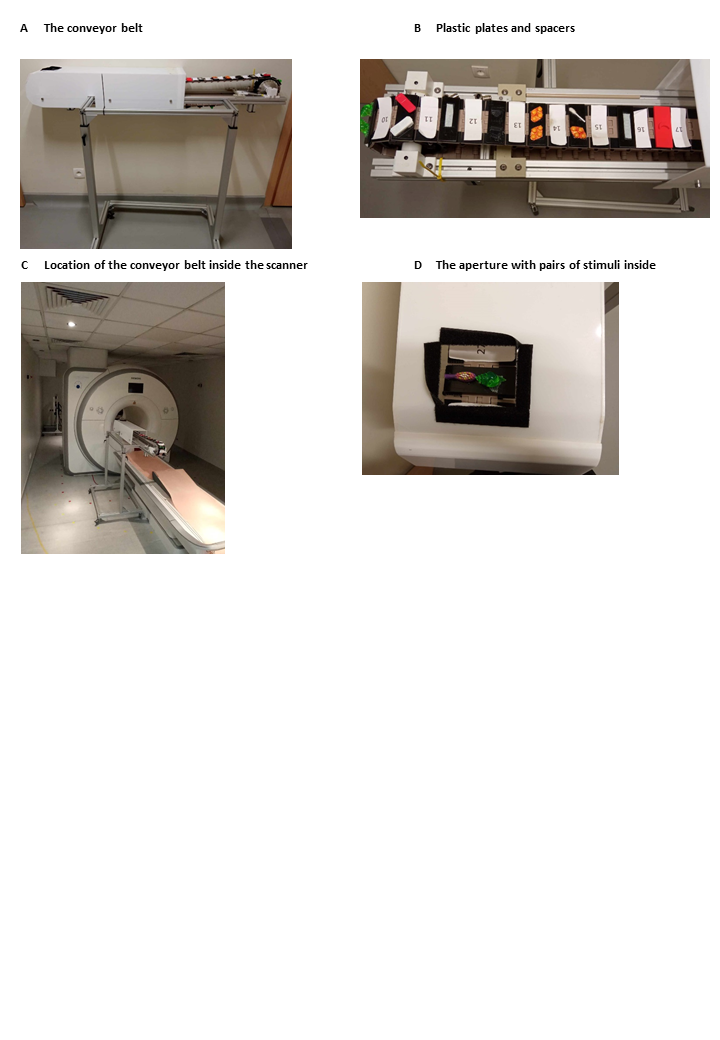

Supplement: Figure 1-1 — (A) The conveyor belt was specially designed for this study to be fMRI-compatible. (B) A picture of the belt with plastic plates containing pairs of stimuli and spacers – gaps between each stimuli plate intended to avoid any possible mistakes. (C) The device was placed above the participants’ thighs on their reading side. The researcher moved the chain and remained at the end of the device. (D)The participants’ reading hand was located in a special aperture, allowing them to touch only one pair of stimuli at a time. Download Figure 1-1, TIF file. [file eneuro-13-ENEURO.0002-25.2025-s002.tif]

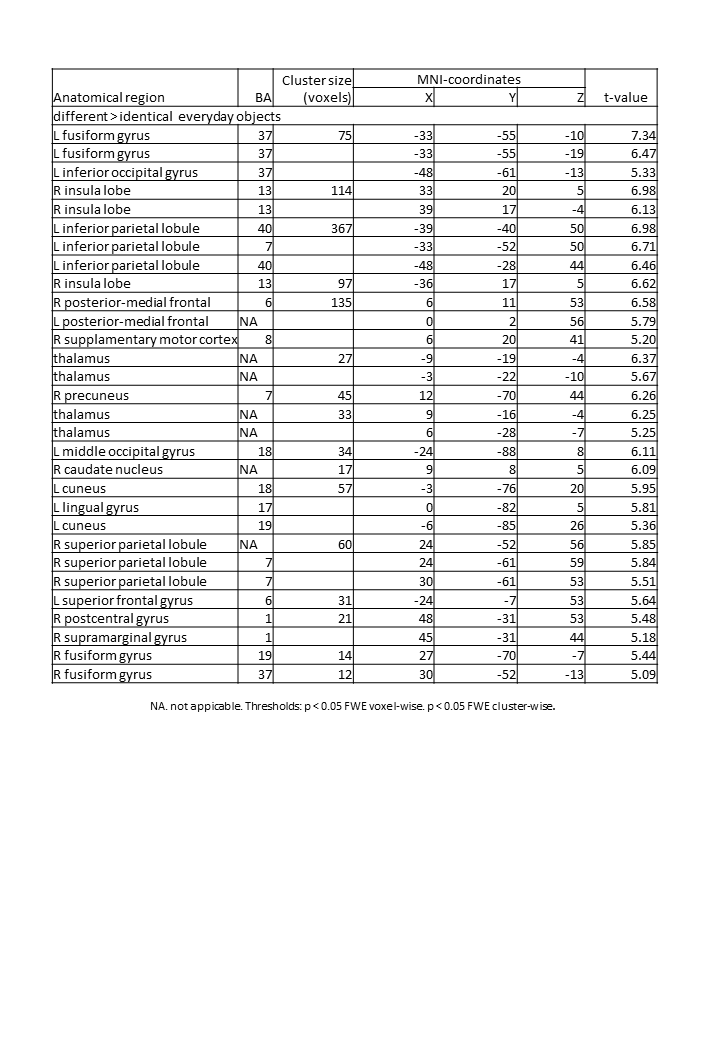

Supplement: Figure 2-1 — Brain activation induced by everyday objects in different > identical condition. Download Figure 2-1, TIF file. [file eneuro-13-ENEURO.0002-25.2025-s003.tif]

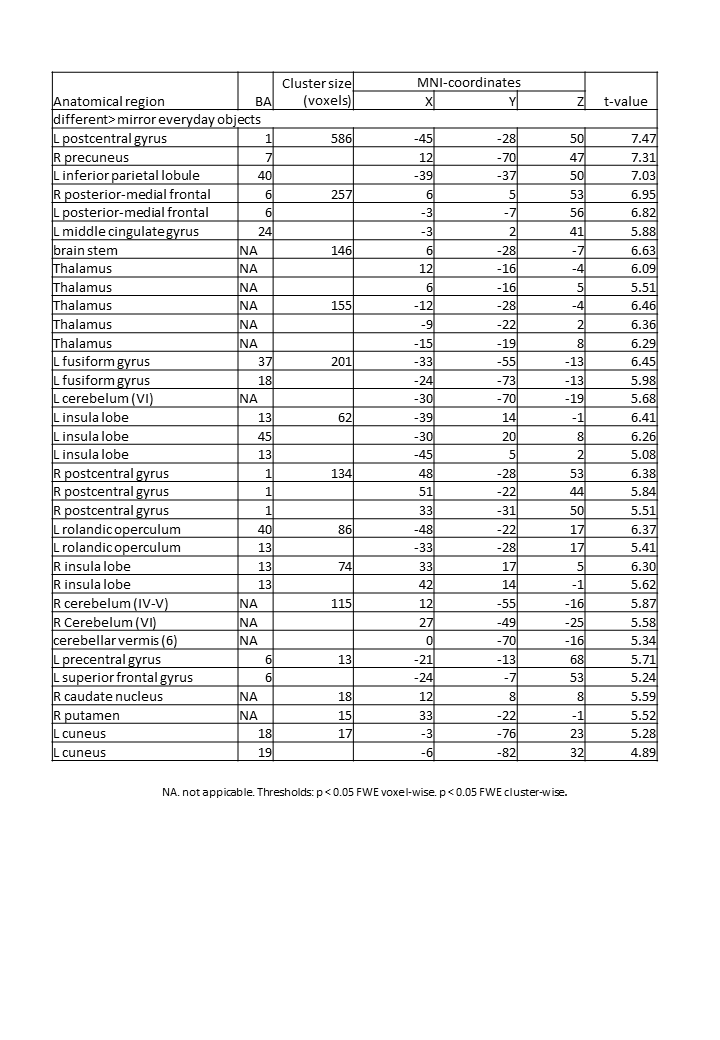

Supplement: Figure 2-2 — Brain activation induced by everyday objects in different > mirror condition. Download Figure 2-2, TIF file. [file eneuro-13-ENEURO.0002-25.2025-s004.tif]

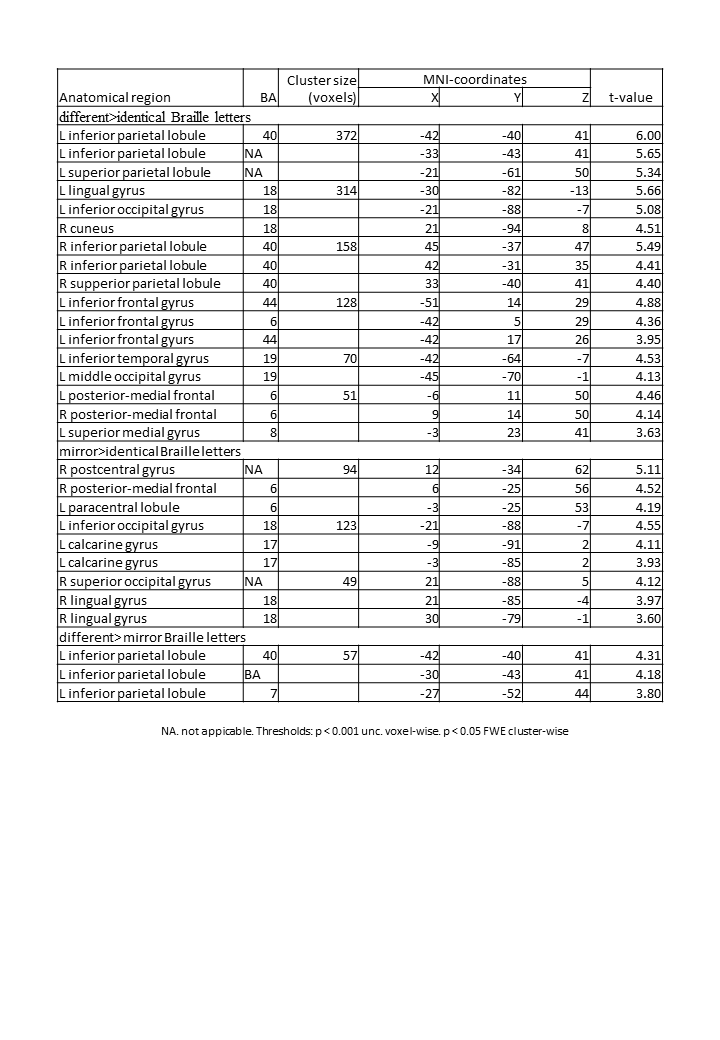

Supplement: Figure 3-1 — Brain activation induced by Braille letters in three conditions: different > identical, mirror > identical, different > mirror. Download Figure 3-1, TIF file. [file eneuro-13-ENEURO.0002-25.2025-s005.tif]

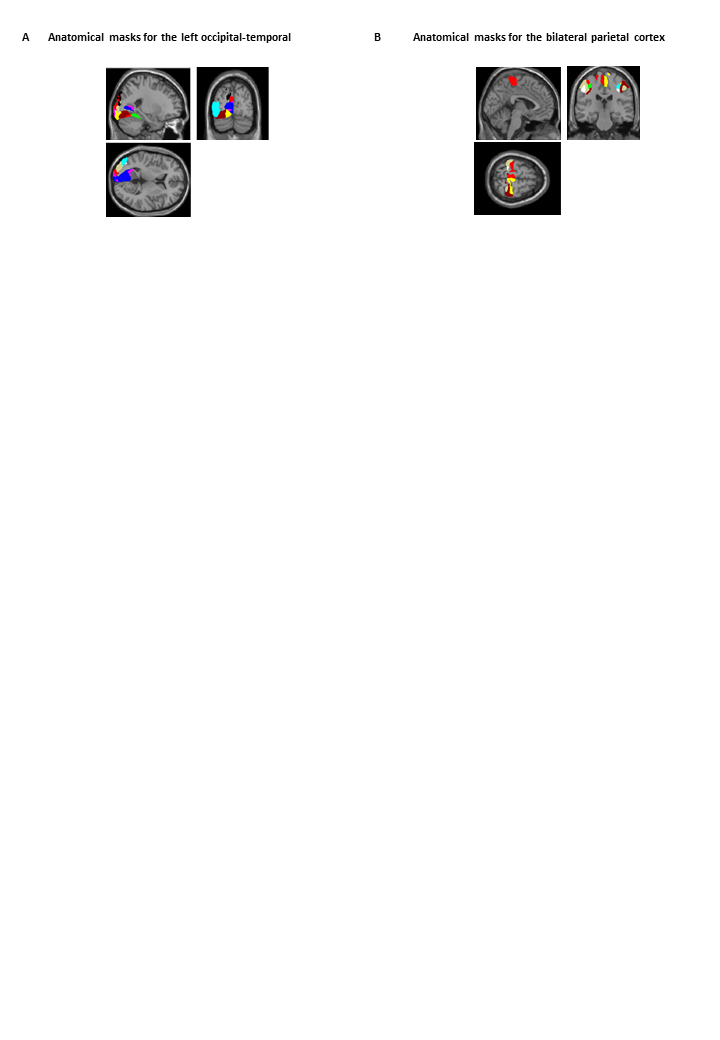

Supplement: Figure 4-1 — (A) Anatomical masks for the left occipital-temporal were created using the SPM Anatomy Toolbox 2.2b (Eickhoff et al., 2005). The left occipital-temporal regions encompassed areas such as 1) the primary visual cortex (V1 and V2 i.e., BA 17 and BA 18), 2) middle-temporal cortex (hOC5 (V5 / MT+))(, 3) ventral (V3v / V4) and 4) dorsal extrastriate cortex (hOC3d / hOC4d), 5) fusiform gyrus (Areas FG1, FG2, FG3 and FG4), and 6) lateral occipital cortex (extrastriate areas hOc4la and hOc4lp). (B) Anatomical masks for the bilateral parietal cortex created using the SPM Anatomy Toolbox 2.2b (Eickhoff et al., 2005) included: 1) the intraparietal sulcus(Areas hIP1, hIP2, hIP3), 2) motor cortex (Areas 4a and 4p), and 3) primary somatosensory cortex (Areas 1, 2, 3a, 3b). Download Figure 4-1, TIF file. [file eneuro-13-ENEURO.0002-25.2025-s006.tif]

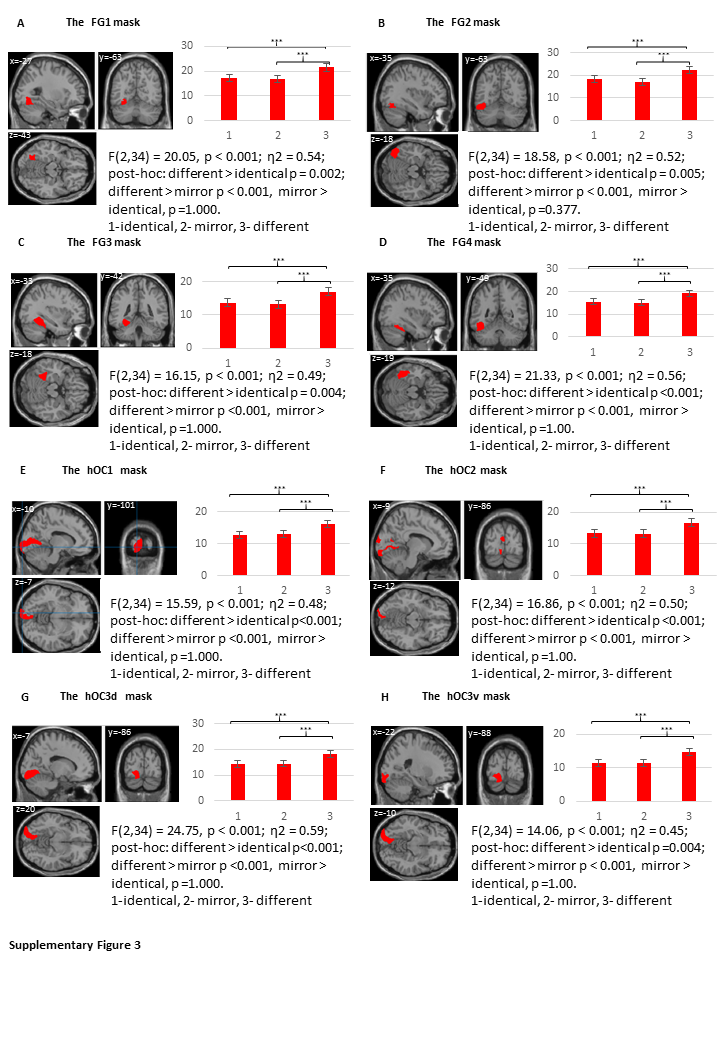

Supplement: Figure 4-2 — (A-H) Anatomical masks for the left occipital-temporal created using the SPM Anatomy Toolbox 2.2b (Eickhoff et al., 2005). The left occipital-temporal regions encompassed areas such as 1) the primary visual cortex (V1 and V2, i.e., BA 17 and BA 18), 2) the middle-temporal cortex (hOC5 (V5 / MT+))(, 3) ventral (V3v / V4) and 4) dorsal extrastriate cortex (hOC3d / hOC4d). All figures present individual results in individual masks for objects. Thresholds levels: *p < 0.05, ** p < 0.01, ***p < 0.001. Error bars represent S.E.M. Download Figure 4-2, TIF file. [file eneuro-13-ENEURO.0002-25.2025-s007.tif]

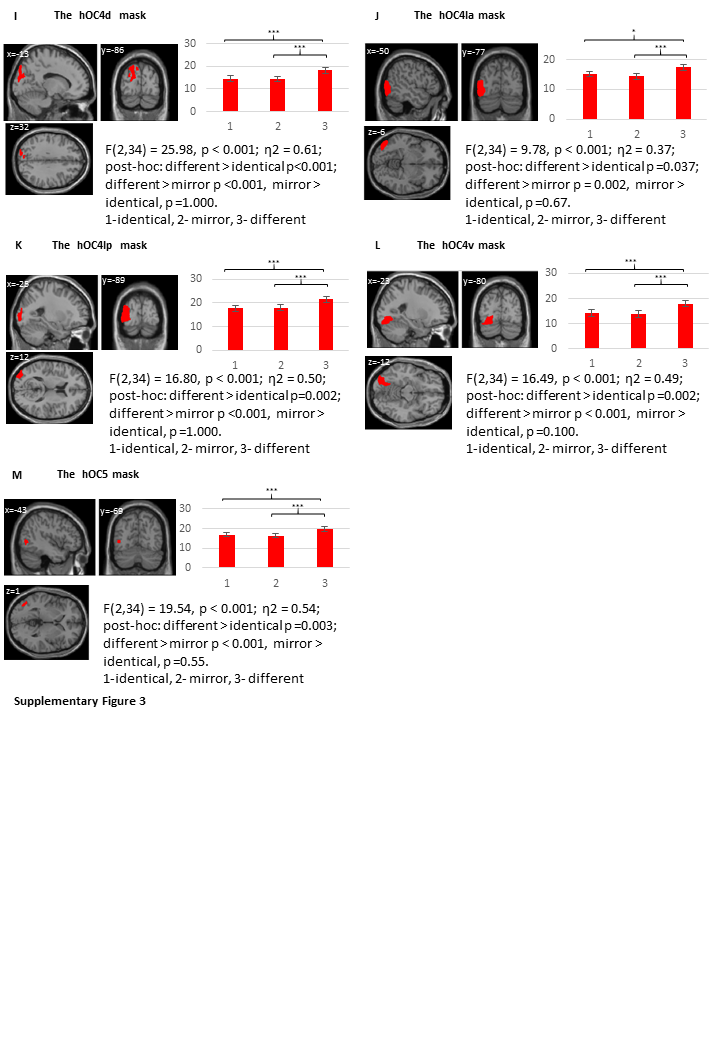

Supplement: Figure 4-3 — (I-M) Anatomical masks for the left occipital-temporal created using the SPM Anatomy Toolbox 2.2b (Eickhoff et al., 2005). The left occipital-temporal regions encompassed areas such as 1) the primary visual cortex (V1 and V2, i.e., BA 17 and BA 18), 2) the middle-temporal cortex (hOC5 (V5 / MT+))(, 3) ventral (V3v / V4) and 4) dorsal extrastriate cortex (hOC3d / hOC4d). All figures present individual results in individual masks for objects. Thresholds levels: *p < 0.05, ** p < 0.01, ***p < 0.001. Error bars represent S.E.M. Download Figure 4-3, TIF file. [file eneuro-13-ENEURO.0002-25.2025-s008.tif]

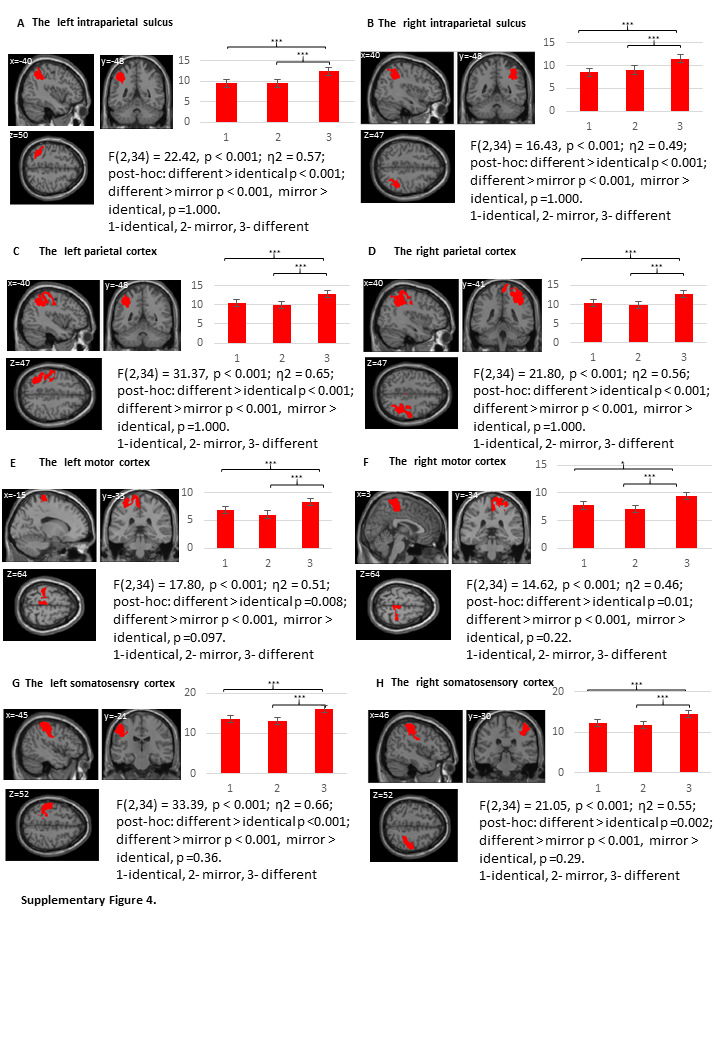

Supplement: Figure 4-4 — (A-H) Anatomical masks for the bilateral parietal cortex created using the SPM Anatomy Toolbox 2.2b (Eickhoff et al., 2005) included: 1) the intraparietal sulcus(Areas hIP1, hIP2, hIP3), 2) motor cortex (Areas 4a and 4p), and 3) primary somatosensory cortex (Areas 1, 2, 3a, 3b). All figures present individual results in individual masks for objects. Thresholds levels: *p < 0.05, ** p < 0.01, ***p < 0.001. Error bars represent S.E.M. Download Figure 4-4, TIF file. [file eneuro-13-ENEURO.0002-25.2025-s009.tif]

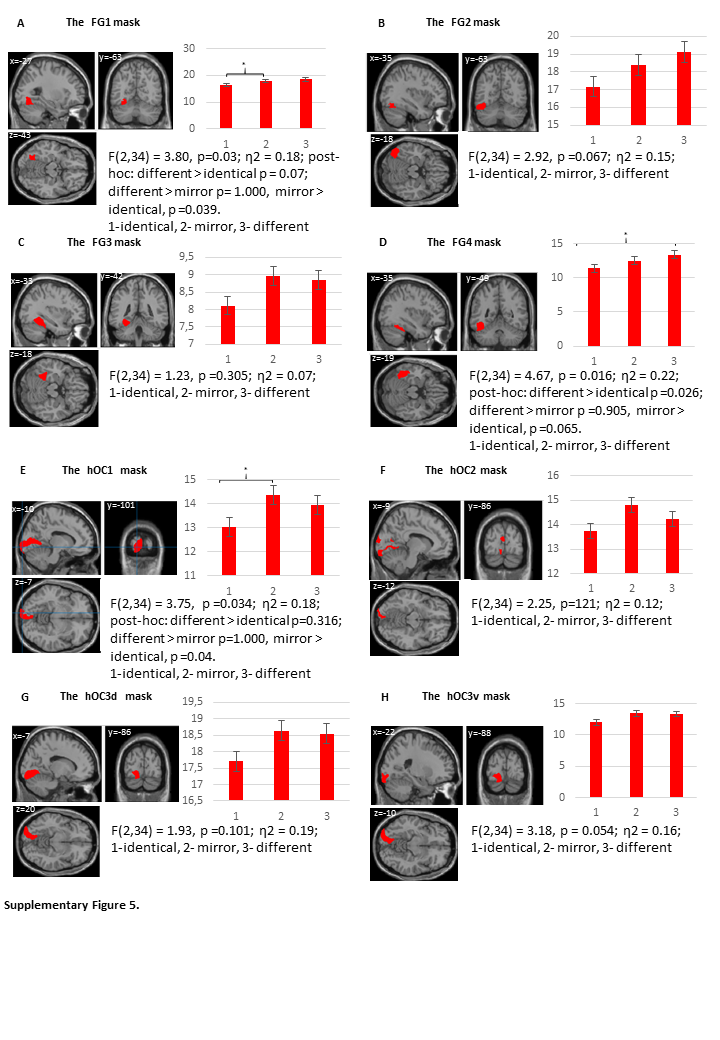

Supplement: Figure 4-5 — (A-H) Anatomical masks for the left occipital-temporal were created using the SPM Anatomy Toolbox 2.2b (Eickhoff et al., 2005). The left occipital-temporal regions encompassed areas such as 1) the primary visual cortex (V1 and V2, i.e., BA 17 and BA 18), 2) the middle-temporal cortex (hOC5 (V5 / MT+))(, 3) ventral (V3v / V4) and 4) dorsal extrastriate cortex (hOC3d / hOC4d). All figures present individual results in individual masks for Braille letters. Thresholds levels: *p < 0.05, ** p < 0.01, ***p < 0.001. Error bars represent S.E.M. Download Figure 4-5, TIF file. [file eneuro-13-ENEURO.0002-25.2025-s010.tif]

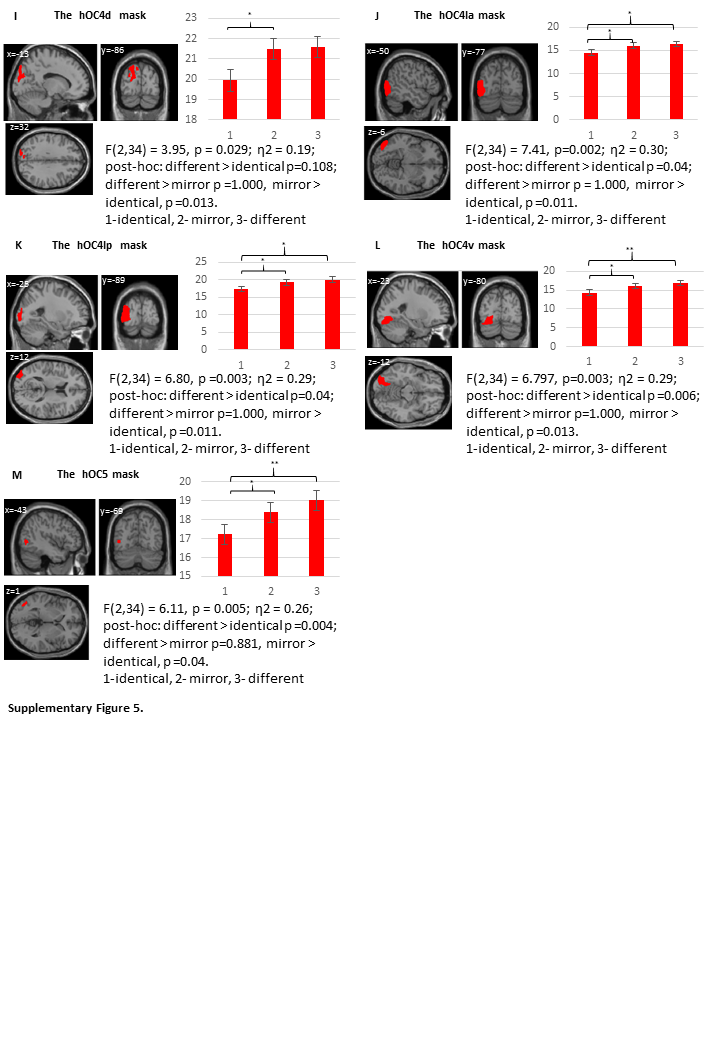

Supplement: Figure 4-6 — (I-M) Anatomical masks for the left occipital-temporal were created using the SPM Anatomy Toolbox 2.2b (Eickhoff et al., 2005). The left occipital-temporal regions encompassed areas such as 1) the primary visual cortex (V1 and V2, i.e., BA 17 and BA 18), 2) the middle-temporal cortex (hOC5 (V5 / MT+))(, 3) ventral (V3v / V4) and 4) dorsal extrastriate cortex (hOC3d / hOC4d). All figures present individual results in individual masks for Braille letters. Thresholds levels: *p < 0.05, ** p < 0.01, ***p < 0.001. Error bars represent S.E.M. Download Figure 4-6, TIF file. [file eneuro-13-ENEURO.0002-25.2025-s011.tif]

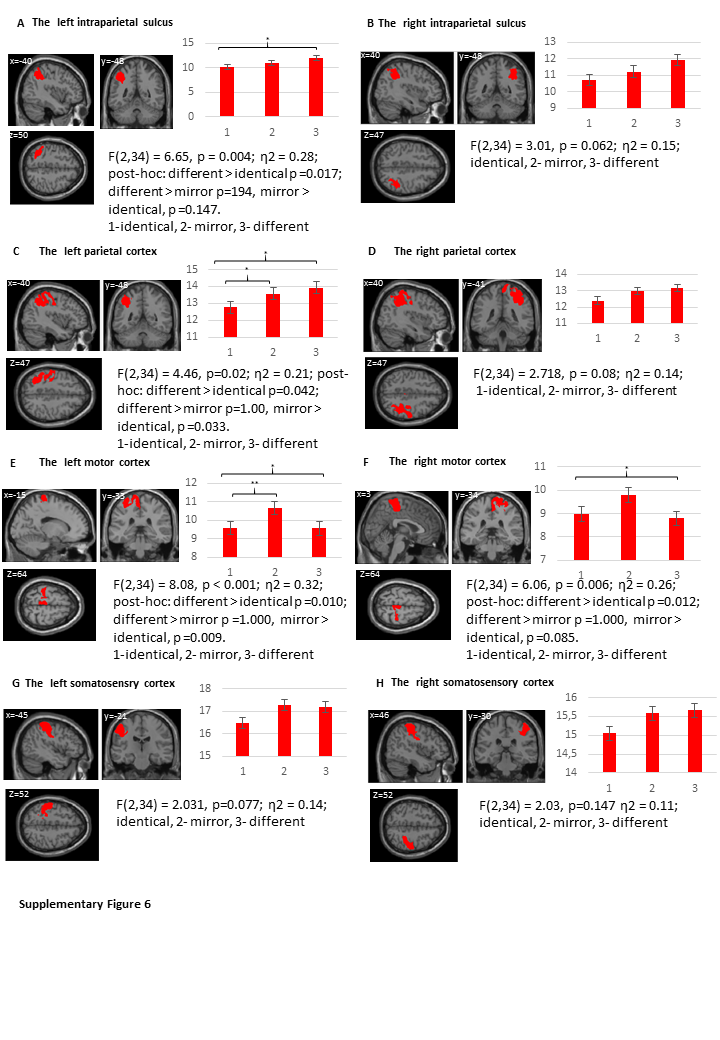

Supplement: Figure 4-7 — (A-H) Anatomical masks for the bilateral parietal cortex created using the SPM Anatomy Toolbox 2.2b (Eickhoff et al., 2005) included: 1) the intraparietal sulcus(Areas hIP1, hIP2, hIP3), 2) motor cortex (Areas 4a and 4p), and 3) primary somatosensory cortex (Areas 1, 2, 3a, 3b). All figures present individual results in individual masks for Braille letters. Thresholds levels: *p < 0.05, ** p < 0.01, ***p < 0.001. Error bars represent S.E.M. Download Figure 4-7, TIF file. [file eneuro-13-ENEURO.0002-25.2025-s012.tif]

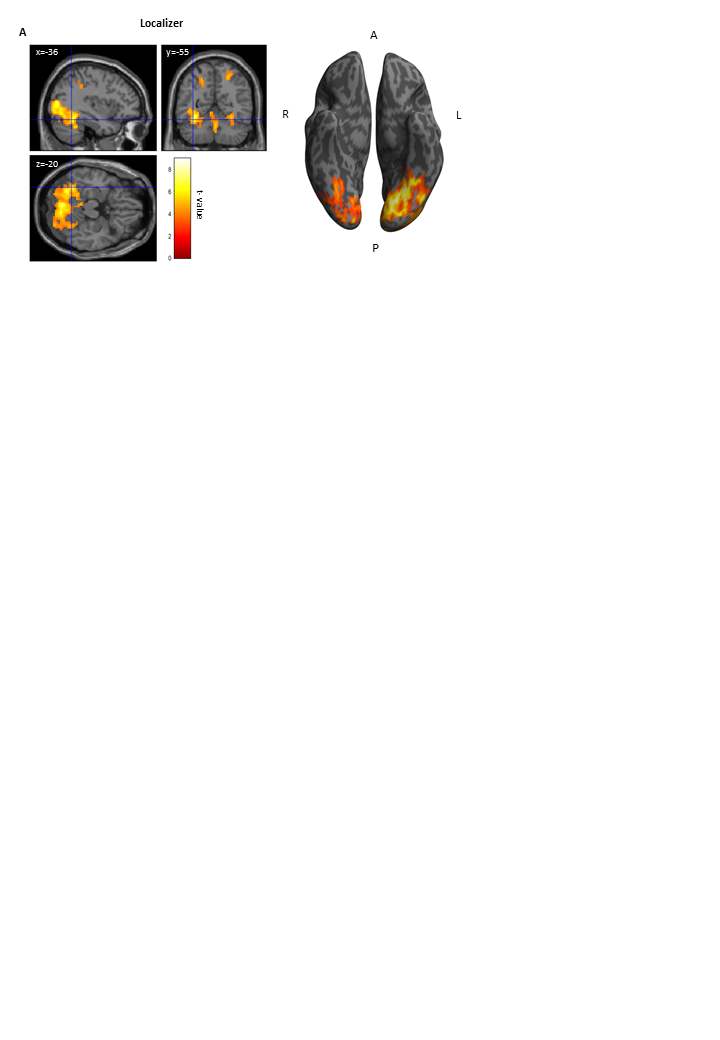

Supplement: Figure 4-8 — Localizer –Experiment 2. The statistical map obtained in the localizer scan. Reading Braille letters activated the typical reading network of the sighted. Thresholds: (A) p < 0.001 unc. voxel-wise, p < 0.05 FWE cluster-wise; Download Figure 4-8, TIF file. [file eneuro-13-ENEURO.0002-25.2025-s013.tif]
